# An Interpretable Fuzzy Distance-Based Ensemble Framework with SHAP Analysis for Clinically Transparent Prediction of Diabetes

**DOI:** 10.3390/diagnostics16091254

**Published:** 2026-04-22

**Authors:** Asif Hassan Syed, Altyeb Altaher Taha, Ahmed Hamza Osman, Yakubu Suleiman Baguda, Hani Moaiteq Aljahdali, Arda Yunianta

**Affiliations:** 1 Department of Computer Science, Faculty of Computing and Information Technology, Rabigh, King Abdulaziz University, Jeddah 21589, Saudi Arabia; shassan1@kau.edu.sa; 2 Department of Information Technology, Faculty of Computing and Information Technology, Rabigh, King Abdulaziz University, Jeddah 21589, Saudi Arabia; 3 Department of Information Systems, Faculty of Computing and Information Technology, Rabigh, King Abdulaziz University, Jeddah 21589, Saudi Arabia; ahoahmad@kau.edu.sa (A.H.O.); ysoleman1@kau.edu.sa (Y.S.B.); hmaljahdali@kau.edu.sa (H.M.A.); ayunianta@kau.edu.sa (A.Y.)

**Keywords:** diabetes prediction, interpretable machine learning, fuzzy fusion, gradient boosting, ensemble learning, eXplainable AI (XAI), SHAP analysis, clinical decision support

## Abstract

**Background/Objectives:** Diabetes is a chronic metabolic disorder affecting global health, where early prediction can significantly reduce disease severity. **Methods:** This research proposes an interpretable multi-metric fuzzy distance-based ensemble (MMFDE) that integrates multi-variant gradient-boosting classifiers (GBM, LightGBM, XGBoost, and AdaBoost) through a novel fuzzy fusion mechanism designed for intrinsic interpretability. Unlike conventional ensembles relying on opaque averaging or voting, MMFDE transforms base classifier predictions into a high-dimensional fuzzy space quantified via a weighted hybrid distance incorporating Euclidean, Manhattan, Chebyshev, and cosine metrics against ideal diabetic and non-diabetic reference vectors. These distances are translated into membership degrees with the help of exponentially decaying functions, which give clinicians calibrated confidence scores for every prediction. Comprehensive SHAP analysis identifies important clinical risk factors (glucose, BMI, and diabetes pedigree function), which show concordance with the medical literature, thereby giving greater clinical trust. **Results:** Experimental evaluations on two publicly available datasets, Hospital Frankfurt Germany Diabetes Dataset (HFGDD) and Pima Indians Diabetes Dataset (PIDD), show that MMFDE outperforms all base models with a significant accuracy of 94.83% and Area Under the Curve (AUC) of 97.66% on HFGDD and three different levels of interpretability: geometric transparency via distance-based decisions, confidence-calibrated uncertainty estimates, and feature-level explanations via SHAP. The confidence thresholds enabled in the framework support risk stratification clinical workflows with high-confidence predictions for automated screening and cases with moderate/low confidence flagged out for review by the clinician. **Conclusions:** By demonstrating that high performance and interpretability need not be mutually exclusive, MMFDE advances trustworthy AI for clinical decision support, addressing the critical need for transparent and clinically actionable diabetes prediction systems.

## 1. Introduction

Diabetes mellitus is a chronic illness that can be severe if not treated. Thus, it can eventually lead to vision impairment, kidney dysfunction, limb amputation, and cardiovascular complications. Diabetes is an important global concern for public health, affecting around 10.5% of the total global population [1]. More importantly, it is expected to rise further due to increasing rates of aging and obesity [2].

Diabetes is generally categorized into two major classes: Type 1 Diabetes Mellitus (T1DM) and Type 2 Diabetes Mellitus (T2DM). T1DM is characterized by the immune-mediated damage of insulin-making units of the pancreas, thus impairing the body’s capacity to produce enough insulin, which leads to a state of higher blood glucose concentration, referred to as hyperglycemia, and in many instances, this condition affects adolescents and teenagers. T2DM is a chronic illness resulting from a combination of genetic susceptibility and lifestyle variables like unhealthy eating habits, insufficient levels of physical exercise, hypertension, and being overweight. Although both T1DM and T2DM are significant illnesses that require continuing care, T2DM is more frequent and may be substantially prevented with early identification and lifestyle modifications [3,4].

Furthermore, diabetic patients often do not show any signs or symptoms during the early phases of the illness; therefore, the early identification of the disease is important for timely diagnosis [5]. Clinical guidelines recommend checking adults who are overweight at any age, and adults of normal weight should be checked starting at 35 years of age [6,7]. Classical diagnostic approaches, such as fasting blood sugar and HbA1c measurements, have limitations in terms of expenses, complexity, and accessibility, and this leads to delayed identification and subsequent complications [8].

Machine learning (ML) methods have demonstrated remarkable effectiveness in creating disease prediction models. Researchers have applied ML to diabetes research, including diagnostic variable identification, disease progression forecasting, complication assessment, and drug discovery [9,10,11,12,13,14,15,16]. The applications of ML are broad, and recent research has shown its success in predicting COVID-19 severity [17,18], detection of tuberculosis from chest X-rays [19], classification of leukemia from microscopic images [20], and management of chronic cardiac diseases using IoT devices [21], highlighting the potential of AI in medicine [22,23]. However, despite high predictive accuracy, the clinical adoption of ML models remains limited due to their “black-box” nature [24]. Clinicians require not only accurate predictions but also transparent, interpretable explanations that align with medical reasoning and support clinical decision-making [25,26].

The interpretability gap in ML-based diabetes prediction presents three critical challenges:

- **Clinical Trust:** Physicians are reluctant to act on predictions they cannot explain, particularly for high-stakes diagnoses [27];
- **Regulatory Compliance:** Healthcare regulations increasingly require algorithmic transparency and accountability [28];
- **Actionable Insights:** Black-box models cannot identify which risk factors drive predictions, limiting preventive intervention opportunities [29].

Ensemble learning reduces these limitations by utilizing a fusion between different models to improve the prediction accuracy. However, conventional fusion strategy usually adopts simple voting or averaging schemas to perform fusion that ignores dependency and interaction between the base classifiers and generates outputs even more difficult to interpret than individual models [30,31,32]. Correlated model errors may lead to compromised predictive capabilities, and a lack of transparency in ensemble decision-making adds to an interpretability issue.

Recent progress in eXplainable AI (XAI) has tried to overcome these difficulties through post hoc explanation techniques, e.g., SHAP (SHapley Additive exPlanations) and LIME (Local Interpretable Model-agnostic Explanations) [33,34]. While these approaches can give feature importance rankings, they consider the model a black box and give explanations that may not accurately reflect the internal decision process of the model [35]. Furthermore, post hoc explanations may also be inconsistent in different methods, as well as fail to capture nuanced interactions in ensemble architectures [36].

There is an increasingly recognized need to ensure interpretability is part of the design of the model, not applied after the model has been built [37]. Intrinsically interpretable models have several benefits: explanations are considered faithful to the actual reasoning of the model, computational overhead is minimized, and clinical trust is increased via transparency [38]. However, current intrinsically interpretable methods often compromise the predictive performance for explainability—this creates a false dichotomy between the accuracy and transparency [39,40].

This paper fills the void between high-performance ensemble learning and clinical interpretability by introducing a multi-metric fuzzy distance-based ensemble (MMFDE), which is intrinsically interpretable with state-of-the-art predictive capability. There are three levels of interpretability provided by our framework:

1. **Geometric Interpretable:** The fuzzy fusion mechanism maps the classifier outputs into a high-dimensional space where distances to ideal reference vectors allow easy geometric explanations to be given for each decision.
2. **Confidence-Calibrated Interpretability:** Fuzzy membership scores act as natural measures of confidence so that clinicians can differentiate between high-certainty and ambiguous cases that need more examination.
3. **Clinical Feature Interpretability:** Comprehensive SHAP analysis can identify key risk factors and help show alignment with known clinical knowledge, and build trust amongst physicians.

Unlike typical ensemble methods based on opaque averaging or voting, in our proposed method, the base classifier predictions will be transformed into a high-dimensional fuzzy space in which the decision boundaries are geometrically interpretable. This transformation is measured through a weighted hybrid distance including Euclidean, Manhattan, Chebyshev, and cosine metrics measured against ideal diabetic and non-diabetic vectors. The multi-metric approach essentially captures complementary aspects of prediction geometry: Euclidean distance measures the overall magnitude difference; Manhattan distance provides the pro-intolerance to the outliers; cosine distance captures the directionality alignment irrespective of the magnitude; and Chebyshev distance seeks the worst-case deviations.

To systematically address the issue of predictive uncertainty, the computed distances are converted into a degree of membership using exponential decay functions to obtain calibrated confidence scores for each prediction. A final and strong inference is obtained with the use of a normalized fuzzy scoring mechanism that reconciles conflicting model outputs while preserving interpretability.

The proposed framework deals with the interpretability challenge by means of both in-built design and thorough post hoc analysis. The fuzzy membership values provide immediate insight into decision confidence, while SHAP analysis delivers detailed feature attribution at both global and local levels. This dual approach ensures that clinicians can understand not only what the model predicts but also why it makes each prediction and which risk factors drive individual patients’ outcomes.

This study makes the following contributions:

1. Proposes an intrinsically interpretable multi-metric fuzzy distance-based ensemble that enhances diabetes prediction accuracy while maintaining decision transparency through geometric interpretability of the fusion mechanism.
2. Introduces a confidence-calibrated fuzzy fusion framework that maps multi-variant gradient-boosting classifier outputs (GBM, LightGBM, XGBoost, and AdaBoost) into a high-dimensional fuzzy space using a weighted hybrid distance (Euclidean, Manhattan, Chebyshev, and cosine) against ideal diabetic and non-diabetic reference vectors, providing clinicians with uncertainty-aware predictions.
3. Provides SHAP-based validation demonstrating that the model-identified risk factors (glucose, BMI, and Diabetes Pedigree Function) align with established clinical knowledge, thereby supporting physician trust and clinical acceptability.
4. Demonstrates through extensive experiments on two publicly available diabetes datasets (HFGDD and PIDD) that the proposed approach not only outperforms base classifiers (94.83% accuracy, 97.66% AUC on HFGDD) but also delivers clinically interpretable explanations suitable for integration into decision support systems.
5. Presents a practical framework for implementing interpretable ensemble methods in the clinical context, as it includes the use of confidence thresholds for identifying cases that are uncertain and feature attribution visualizations for each patient consultation.

The rest of this paper is organized as follows: Section 2 is a review of the previous research efforts in diabetes prediction and interpretable machine learning. Section 3 explains the proposed MMFDE framework, focusing on its interpretability features. Experimental results comprising global SHAP analysis results and clinical interpretation are presented in Section 4. Section 5 is a discussion of clinical implications as well as considerations for deployment. Section 6 concludes our work and offers aspects of future research.

## 2. Related Work

This section provides an overview of the existing literature for three key areas: (1) conventional methods in machine learning for diabetes prediction, (2) ensemble methods and their application to diabetes, and (3) recent developments in interpretable and explainable AI for healthcare applications. For each category, we critically evaluate both predictive performance and the extent to which methods provide clinically useful explanations.

### 2.1. Machine Learning for Diabetes Prediction

Several studies utilized ML schemes to predict diabetes. For example, Gupta et al. [29] suggested an advanced neural network (NN) method for the classification of diabetes based on a dataset of 768 cases with nine features. They additionally performed a comparative assessment between traditional ML and ensemble learning methods for forecasting diabetes. Hyperparameter tuning was employed to optimize their method. The findings demonstrated that the suggested method surpassed the alternative methods with an accuracy score of 84.42%, specificity of 94.11%, recall of 65.40%, and precision of 85.12%. However, the authors did not address model interpretability, limiting clinical applicability.

In [30], Bhoi evaluated different supervised machine learning algorithms on the Indian PIMA dataset. The Orange platform 3.29 and Python 3.8 libraries were utilized for conducting experiments. Logistic regression performed better in comparison to alternative models and attained the best accuracy of 76.80%. Qin et al. [31] used CATBoost, Logistic Regression (LR), Support Vector Machine (SVM), XGBoost, and Random Forest (RF) methods for diabetes forecasting. The best result was achieved by CATBoost with an accuracy of 82.1% and an AUC of 83%. While these studies demonstrate predictive capability, they treat models as black boxes without explanation mechanisms.

Kalagotla et al. [32] proposed a stacking-based method for improving the classification of the PIMA Indian diabetes data set. The approach integrates a multi-layer perceptron, logistic regression, and a support vector machine as the base learners. The results obtained from the experiments show that the heterogeneous stacking ensemble (78.2%) is more accurate than the homogeneous AdaBoost (76.54%) by 1.66%. Despite improved performance, the stacked architecture compounds interpretability challenges, as final predictions aggregate multiple opaque models.

Chang et al. [41] trained and evaluated three supervised machine learning methods, namely J48 Decision Tree (DT), Naive Bayes (NB), and RF-based on the Pima dataset. The efficiency of each of the algorithms is evaluated to determine which algorithm is most accurate, precise, sensitive, and specific. The best accuracy of 79.13% was obtained by using the random forest algorithm. Ramesh et al. [42] presented a method that uses the SVM algorithm for predicting diabetes using the Pima database. They utilized procedures such as attribute scaling, augmentation, imputation, and selection. Their proposed scheme attained an accuracy, specificity, and sensitivity of 83.20%, 79%, and 87.20%, respectively, indicating consistent and reliable performance when examined through 10-fold stratified cross-validation.

The PIMA dataset published in the Kaggle repository was used by Ahmed et al. [43] in their research work. They used exploratory data analysis (EDA) to identify the interactions among multiple attributes in the dataset through graphical visualization. Four ML techniques (LR, DT, NB, and RF) were developed using Python to predict the presence of diabetes in the PIMA dataset. Findings of the study indicated that RF is more accurate (80%), precise (82%), and sensitive (88%) than the other methods, LR, NB, and DT.

Butt et al. [44] integrated an IoT-based observing technique that enables real-time blood sugar measurement with an ML architecture for diabetes forecasting and diagnosis. They used RF, LR, MLP, and long short-term memory (LSTM) classifiers to predict diabetes employing the benchmark PIMA dataset. The LSTM and MLP classifiers obtained accuracies of 87.26% and 86.08%, respectively, which demonstrates the usefulness of the suggested scheme in healthcare-related prediction tasks.

Mansouri et al. [45] explored the application of the k-nearest neighbors (KNN) algorithm for diabetes prediction based on the Pima dataset. The study concentrated on the classification of individuals into non-diabetic and diabetic classes and analyzing the impact of different factors on predictive achievement. The authors followed standard data preprocessing techniques such as missing value treatment, normalization of the features, and the splitting of the data into testing and training data. The KNN algorithm was then optimized and evaluated using the best settings. Findings of experiments showed that the prediction accuracy of this suggested approach is 76%.

Iparraguirre-Villanueva et al. [46] presented the implementation of several classical ML algorithms, including KNN, LR, DT, NB, and SVM, to predict diabetes based on the Pima Indian datasets. Their experimental results revealed that KNN and NB achieved superior performance to the alternative methods with an accuracy of 79.6% and 77.2%, respectively.

Despite achieving respectable accuracies, all the studies share a common limitation: they prioritize predictive performance while neglecting interpretability. None offer ways to allow clinicians to better understand why certain predictions are made, which features the predictions are based on, or how confident the model is in cases where the predictions are ambiguous. This “black-box” approach represents a huge obstacle to clinical implementation [39].

### 2.2. Ensemble Methods in Disease Prediction

Ensemble learning has become a powerful technique to enhance the predictive performance by combining several models [40]. Recent reviews from Almulihi et al. [24] and Mahajan et al. [25] show that ensembles have been consistently shown to be more accurate than individual classifiers in a variety of disease prediction tasks.

Common ensemble approaches involve:

- **Bagging methods** (e.g., Random Forest), which use bootstrap samples to train models and aggregate them using voting [47].
- **Boosting Methods** (e.g., AdaBoost and Gradient Boosting) that successively correct predecessor errors [48].
- **Stacking methods** that train a meta-learner to run on the outcomes of base models [49].
- **Voting ensembles** that combine predictions through simple or weighted averaging [26,27,28].

Livieris et al. [26,27] demonstrated weighted voting ensembles for medical imaging classification, while Majumder et al. [28] applied hybrid ensemble classifiers for heart disease prediction. However, these conventional mechanisms for fusion have two limits with regard to interpretability:

First, simple averaging or voting hides how much each of the models contributed, and gives no understanding about why the ensemble arrived at a certain decision [50]. Second, when the base models disagree, standard aggregation methods cannot indicate which prediction is to be trusted and why the conflict occurred [51].

Recent efforts to address ensemble interpretability include:

- **Rule-based ensembles** that combine interpretable IF-THEN rules [52];
- **Attention-based ensembles** that weight models based on input characteristics [53];
- **Explainable boosting machines** that sustain additivity for interpretability [54].

However, these approaches often compromise predictive performance to be interpretable or provide sufficient explanations that are not sufficiently granular to assist clinical decision making [55].

### 2.3. Explainable AI (XAI) in Healthcare and Diabetes Prediction

The increasing awareness of the importance of ensuring that clinical AI systems are interpretable has spurred the development of research into eXplainable AI (XAI) for healthcare [56]. The XAI methods generally have two categories.

#### 2.3.1. Post Hoc Explanation Methods

Post hoc methods are methods used to explain black-box models after their training by analyzing their input–output relationships [57]. The most popular methods adopted are:

- **SHAP (SHapley Additive exPlanations):** Based on cooperative game theory, SHAP assigns each feature an importance value for a particular prediction [33]. SHAP has been used for diabetes prediction to identify important risk factors [58].
- **LIME (Local Interpretable Model-agnostic Explanations):** LIME approximates the local decision boundary with an interpretable surrogate model [34].
- **Partial Dependence Plots (PDP) and Individual Conditional Expectation (ICE):** These visualize how predictions change as features vary [38].
- **Counterfactual Explanations:** These identify minimal feature changes that would alter a prediction [59].

Stiglic et al. [40] thoroughly reviewed the methods of interpretability for healthcare predictive models, pointing out that while post hoc methods offer valuable insights, they come with serious limitations: explanations might not capture the true reasoning of the model [35], different methods can yield conflicting explanations [36], and computational overhead can be significant [38].

#### 2.3.2. Intrinsically Interpretable Models

An alternative paradigm calls for the design of interpretability into models right from the start [37]. Rudin [35] argues that for high-stakes decisions such as medical diagnoses, “black-box models solved with post hoc explanations” should be avoided in favor of intrinsically interpretable models that are “explainable by design.”

Intrinsically interpretable approaches are:

- Linear models and logistic regression with sparse regularization [60].
- Decision trees and rule-based systems that provide explicit decision paths [52].
- Generalized additive models (GAMs) that maintain additivity while capturing nonlinearity [54].
- Attention-based deep learning where attention weights indicate input relevance [53].

For diabetes prediction, intrinsically interpretable models have shown promise but often underperform compared to black-box ensembles [61]. This leads to a perceived trade-off between the accuracy and the interpretability that limits the adoption in clinical implementation.

#### 2.3.3. XAI Applications in Diabetes

A few recent studies used XAI techniques to predict diabetes:

- **Islam et al. [58]** applied SHAP using gradient boosting models and found glucose, BMI, and age to be the dominant factors in diabetes risk prediction. However, their ensemble continued to be a black box as SHAP applied post hoc.
- **Kopitar et al. [62]** compared local explanation methods for diabetes prediction, discovering that different methods created different feature importance rankings, emphasizing the need for explanation consistency.
- **Ellahham [63]** examined the topic of AI interpretability in diabetes management, concluding that the “clinician trust remains the primary barrier to adoption” and stressing the need for models that demonstrate inherent transparency.
- **Deng et al. [64]** proposed an interpretable deep learning model for diabetes prediction based on attention mechanisms with an accuracy of 78.5% and provided feature importance weights.

Despite these improvements, current methods either: (a) provide post hoc explanation to black box ensembles without addressing the inherent opacity of the models, (b) sacrifice predictive accuracy for interpretability, or (c) provide clinically un-actionable explanation [65].

To provide a clear summary of the representative works discussed above, Table 1 presents the key studies in diabetes prediction, their methodologies, performance, and limitations.

A significant gap that still exists in the literature is the tradeoff between predictive performance and interpretability, as summarized in Table 1. Although black box ensembles (e.g., [29,44]) are highly accurate, they are not transparent, and post hoc explanations (e.g., [47]) do not necessarily honor the reasoning behind the model. On the other hand, models that can be interpreted intrinsically (e.g., [52]) tend to trade off predictive accuracy. This article suggests a Multi-Metric Fuzzy Distance-Based Ensemble (MMFDE) to fill this gap outright by creating interpretability to the fusion mechanism without compromising competitive predictive performance.

### 2.4. Research Gap and Positioning of Current Work

As outlined in Table 1 (see above), most existing diabetes prediction studies focus on predictive performance but lack explainability and actionability in clinical practice. To provide a clearer picture of the interpretability features of some of these studies and to highlight the contribution of this study, Table 2 outlines the interpretability features of a number of studies.

The literature review shows several critical gaps that motivate this research:

1. **Interpretability-Accuracy Trade-off:** Existing methods either yield high accuracy with no interpretability (black-box ensembles) or give interpretability with poor accuracy (simple models). There is a need for approaches that preserve ensemble-level performance and provide clinically meaningful explanations.
2. **Explanation Faithfulness:** Post hoc explanations applied to black-box ensembles may not accurately reflect model reasoning, potentially misleading clinicians [51]. Intrinsically interpretable fusion mechanisms are needed.
3. **Confidence Calibration:** Current approaches do not typically include estimates of uncertainty or confidence measures in individual predictions, which reduces their usefulness in clinical decision-making, where when to believe (or not believe) a prediction is so important [66].
4. **Multi-level Interpretability:** Clinicians need explanations at more than one level: global feature importance to understand the risk factors of the disease, local explanations on individual patients, and measures to express the confidence of the decision [67]. Few existing approaches can offer all three.
5. **Clinical Alignment:** Model explanations should also be in line with known medical knowledge, which builds trust. Matching the model-identified risk factors to the clinical literature is an essential but rarely performed task [68].

The proposed Multi-Metric Fuzzy Distance-Based Ensemble (MMFDE) addresses these gaps by:

- Designing interpretability into the fusion mechanism through geometric distance calculations that are inherently transparent.
- Providing calibrated confidence scores via fuzzy membership values that indicate prediction certainty.
- Delivering multi-level interpretability through fuzzy membership (local confidence), SHAP analysis (global/local feature importance), and geometric interpretation (decision boundaries).
- Validating clinical alignment by comparing SHAP-identified features with established diabetes risk factors from medical literature.
- Maintaining competitive predictive performance while achieving transparency, demonstrating that accuracy and interpretability need not be mutually exclusive.

## 3. Interpretable MMFDE Framework

**Figure 1:**
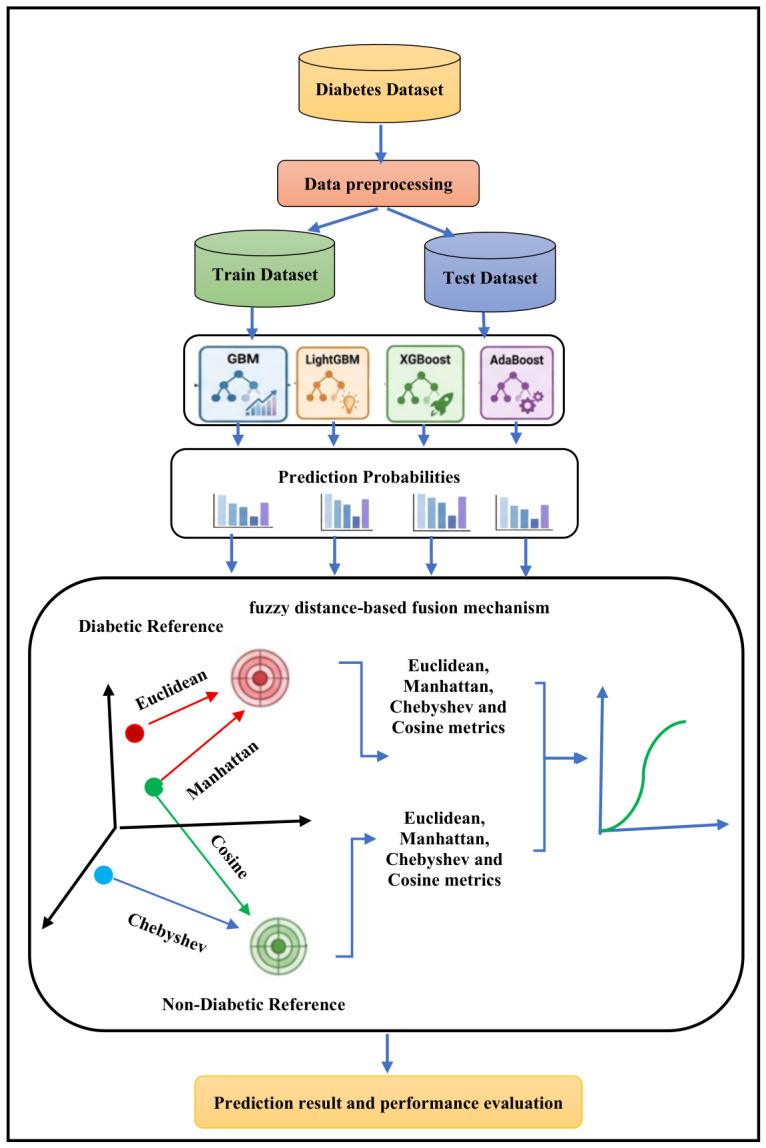
Framework of the proposed interpretable multi-metric fuzzy distance-based ensemble for diabetes prediction, showing the three levels of interpretability: geometric (distance metrics), confidence-based (fuzzy membership), and clinical feature (SHAP analysis).

This part introduces the proposed interpretable multi-metric fuzzy distance-based ensemble framework for enhancing the accuracy of diabetes prediction and ensuring decision transparency and clinical interpretability. The framework includes three integrated elements: (1) systematic preprocessing of data, (2) multi-variant classifiers based on gradient boosting, and (3) a novel fuzzy fusion mechanism that is intrinsically interpretable by design. As opposed to traditional black box ensembles, our framework converts the predictions given by the base classifiers into a high-dimensional fuzzy space in which not only are decision boundaries geometrically interpretable, but confidence scores are calibrated in a natural way, and feature contributions can be rigorously analyzed via SHAP. The interpretability pathways of the complete MMFDE framework are shown in Figure 1.

### 3.1. Datasets and Data Preprocessing

- **Datasets:** The experimental phase of this study is conducted using two different benchmarks: the HFGDD [69], which contains a record of 2000 subjects, and the PIDD [70], which is a collection of 768 patient profiles. The HFGDD was obtained from patients at the outpatient clinic of a university hospital in Frankfurt, Germany, from 2018 to 2020. It comprises 2000 adult patients (1080 women, 920 men; age 54.3 ± 12.7 years) with available clinical records. Diabetes was defined according to the American Diabetes Association (ADA) criteria: fasting plasma glucose ≥126 mg/dL or HbA1c ≥ 6.5% or previous diagnosis by a physician. There are 1120 non-diabetic (56%) and 880 diabetic (44%) subjects. Both datasets have a consistent composition of nine clinical attributes. The target variable is binary type, i.e., a value of “0” indicates non-diabetes and “1” indicates the presence of the disease. A detailed breakdown of these data characteristics can be found in Table 3.
- **Preprocessing for Interpretability:** The datasets are preprocessed by scaling all numerical features in the range of [0, 1] using the min-max normalization procedure [47]. This normalization is important for interpretability, as it allows comparisons of distances in different features with different physical units to have meaningful results (e.g., glucose in mg/dL vs. age in years). The value of the dependent variable was set at 0 for non-diabetic patients and 1 for diabetic patients. A five-fold cross-validation approach preserves class proportions because of class imbalance in a dataset. In each of the iterations, four folds are used for training and one fold for testing, and they are rotated five times to finish the cycle. This validation approach has shown strong performance in diabetes research [71] and can guarantee that interpretability analyses are stable on different partitions of the data. This 5-fold cross-validation was used for all the hyperparameter tuning of the base classifiers and the fuzzy fusion weights, only on the training set. To avoid leakage of information, min–max scaling parameters were calculated on the training folds and applied to the validation folds. The model was tested on the test set (80% training, 20% test) that was not used in the tuning and optimization process.

### 3.2. Gradient Boosting Machine (GBM)

Gradient Boosting Machine (GBM) is a tree-based learning method to construct predictive models by combining several decision trees in a sequential fashion [48]. Every tree is developed to fix the errors of the previously built trees, with more emphasis on the samples that are difficult to predict. From an interpretability perspective, GBM offers feature importance rankings based on the frequency of their usage for splitting, but this is a global approximation and not instance-specific explanations.

### 3.3. LightGBM (Light Gradient Boosting Machine)

LightGBM is a gradient boosting decision tree (GBDT) algorithm, introduced in 2017, which is appreciated for its computational efficiency [72,73]. LightGBM uses a histogram approach with binning of the continuous features, which significantly improves the training time. This histogram strategy has a regularization effect by decreasing model variance, as the grouping of similar feature values reduces the complexity of trees and could reduce overfitting caused by a fine-grained data pattern [74]. LightGBM’s built-in feature importance can be extracted; however, like GBM, it has no local interpretability of individual predictions.

### 3.4. XGBoost (Extreme Gradient Boosting)

XGBoost is a supervised learning algorithm that builds an ensemble of decision trees using gradient boosting with second-order optimization [75,76]. Each successive tree minimizes the residuals of previous trees. XGBoost offers several importance metrics (weight, coverage, and gain) to aid in global feature analysis, but is obscure at the instance level without post hoc explanation techniques.

### 3.5. Adaptive Boosting (AdaBoost)

AdaBoost is a combination of several weak learners with iterative modification of the sample weights, giving more attention to misclassified instances [77]. While the way that AdaBoost updates the weights sequentially does give some hint to the characteristic samples that were hard to classify, the final ensemble selection combination removes any individual decision paths.

All four base models, despite their predictive strength, have a common limitation: at best, they give global feature importance without local explanations for individual predictions or measures of how confident they are in their predictions. This is motivation for our fuzzy fusion mechanism, which is intended to be intrinsically interpretable and, in the meantime, uses the power of these multi-variant classifiers for a prediction.

For the sake of completeness and reproducibility, Table 4 lists the most important hyperparameters of the base classifiers. Other parameters were left with their default values in the libraries (scikit learn, XGBoost, and LightGBM). We used a fixed random seed (42) for all models to ensure reproducibility of the results.

These settings provide complete information about model training and supplement the descriptions in Section 3.2, Section 3.3, Section 3.4 and Section 3.5.

### 3.6. The Interpretable Multi-Metric Fuzzy Distance-Based Ensemble

The proposed fuzzy distance-based ensemble is specifically designed with a view to interpretability at multiple levels. It minimizes the gap between model and ideal results with geometric transparency, confidence calibration, and feature-level explanations. The framework maps the prediction of each classifier into fuzzy space, by means of the weighted combination of Euclidean [78], Manhattan [79], Chebyshev [80], and Cosine distances [81] from the reference points of ideal diabetic and non-diabetic profiles. These distances are then transformed into confidence scores by using an exponential decay function. A final normalized scoring is then used to reconcile conflicting predictions to produce a strong outcome.

It reduces the difference between model outputs and ideal results while providing geometric transparency, confidence calibration, and feature-level explanations. The framework maps each classifier’s prediction into a fuzzy space using a weighted combination of Euclidean [78], Manhattan [79], Chebyshev [80], and Cosine distances [81] from ideal diabetic and non-diabetic reference points. These distances are converted into confidence scores using an exponential decay function. A final normalized scoring process then reconciles conflicting predictions to produce a robust outcome.

The interpretability of our approach is derived from three basic design principles:

1. **Geometric Interpretability:** As distances to ideal reference vectors are being measured in a multi-metric space, the understanding of every prediction can be done in terms of distances from prototypical diabetic and non-diabetic patterns.
2. **Confidence Calibration:** Fuzzy membership values are natural and give confidence measures that help clinicians to differentiate high-certainty cases from ambiguous cases.
3. **Multi-level Explanation:** The framework can provide both global analysis, which uses SHAP, and local explanations (through distance vectors and membership scores).

#### 3.6.1. Mathematical Formulation

Let $X=\left\{\left(x_{1},y_{1}\right),\left(x_{2},y_{2}\right),\ldots ,\left(x_{m},y_{m}\right)\right\}$ denote the dataset, where $x_{i}\in R^{n}$ represents the ith sample and $y_{i}\in \left\{1,2,\ldots ,c\right\}$ is its corresponding class label. Let $S_{z}^{j}\left(x_{i}\right)$ denote the confidence score assigned to sample $x_{i}$ for class j by the zth classifier, where z=1,2,…,M.

For every instance $x_{i}$ and class j, an ensemble representation is constructed by measuring the difference between the ideal confidence vector $1=\;{\left\{1\right\}}_{i=1}^{M}$ and the vector of classifier confidences, defined as $P_{j}(x_{i})=(1-S_{1}^{j}(x_{i}),1-S_{2}^{j}(x_{i}),\ldots ,1-S_{M}^{j}(x_{i})).$

**Interpretability Insight:** This formulation measures how far away the prediction of each classifier is from a prediction that holds full confidence for class *j*. Fewer deviations mean higher alignment with that class. While the current choice of ideal vectors as geometric extremes [1, 1, 1, 1] and [0, 0, 0, 0] provides a simple and interpretable anchor, future work could explore data-driven reference vectors (e.g., class-conditional mean probabilities) to further align with empirical classifier behavior.

Four distance measures are used to calculate separate ensemble representations:

- **Euclidean Distance:** Measures the overall magnitude of deviation and is denoted as $P_{j}^{E}(x_{i})$ and is mathematically computed as follows:
  (1) $$P_{j}^{E}\left(x_{i}\right)=\;\sqrt{\sum_{z=1}^{M}{\left(1-\;S_{z}^{j}(x_{i})\right)}^{2}}$$
  *Interpretation:* Euclidean distance gives a global measure of how far the prediction vector is from the ideal, is sensitive to great deviations in any classifier.
- **Manhattan Distance:** Sum of absolute deviations and are denoted as $P_{j}^{M}(x_{i})$ and are calculated using Equation (2) as follows:
  (2) $$P_{j}^{M}\left(x_{i}\right)=\sum_{z=1}^{M}\left|1-S_{z}^{j}(x_{i})\right|$$
  *Interpretation:* Manhattan distance is more robust for outliers and offers the same sensitivity for any classifiers suitable for situations with several medium disagreements.
- **Cosine Distance:** Captures the directional alignment and is represented as $P_{j}^{C}\left(x_{i}\right)$ and are mathematically computed as follows:
  (3) $$P_{j}^{C}\left(x_{i}\right)=1-\;\frac{\sum_{z=1}^{M}S_{z}^{j}(x_{i})}{\sqrt{\sum_{z=1}^{M}{\left(\;S_{z}^{j}(x_{i})\right)}^{2}\cdot \sqrt{M}}}$$

This is the standard cosine distance between the prediction vector $\left[S_{1}^{j},S_{2}^{j},\ldots ,S_{M}^{j}\right]$ and the ideal vector $\left[1,1,\ldots ,1\right]$, where $\sqrt{M}$ is the Euclidean norm of the ideal vector.

*Interpretation:* Cosine distance is a measure of the similarity of patterns that does not take magnitude into account—which is important in situations where classifiers have a relative confidence but are on different scales.

- **Chebyshev Distance:** Maximum deviation and is represented by $P_{j}^{Ch}(x_{i})$ and calculated mathematically as follows:
  (4) $$P_{j}^{Ch}\left(x_{i}\right)={max}_{z=1,\ldots ,M}\left|1-\;S_{z}^{j}(x_{i})\right|$$
  *Interpretation:* Chebyshev distance finds the disagreeing classifiers in worst-case scenarios, thereby putting into the foreground any potential conflicts within the model that would merit clinical attention.

**Why Multiple Metrics?** Each distance measure confounds the idea of a unique measure of prediction geometry. Euclidean and Manhattan are used to calculate the overall magnitude of deviation; cosine is used to calculate directional agreement; and Chebyshev is used to find extreme outliers. The combination ensures that no one aspect has either a higher or lower weighting, and the weighted average is a comprehensive measure of similarity and is therefore more interpretable than any one metric alone.

For each of the classes j=1,2,…,c, the distances are combined using a weighted average:

(5) $$\Lambda_{j}\left(x_{i}\right)=w_{E}P_{j}^{E}\left(x_{i}\right)+w_{M}P_{j}^{M}\left(x_{i}\right)+w_{C}P_{j}^{C}\left(x_{i}\right)+w_{Ch}P_{j}^{Ch}\left(x_{i}\right)$$

where $w_{E},w_{M},w_{C},\;and\;w_{Ch}$ are the weights given to the Euclidean distance, Manhattan distance, Cosine distance, and Chebyshev distance, respectively. The optimization procedure in finding these weights is described in Section 3.6.2.

The final class is then selected using the minimum rule: we select the class with the smallest combined distance score:

(6) $${\hat{y}}_{i}=arg\;\underset{j}{min}\{\Lambda_{j}(x_{i})\}.$$

Here, ${\hat{y}}_{i}$ signifies the predicted label of sample $x_{i}$.

When the weighted hybrid distance $\Lambda_{j}(x_{i})$ is calculated for each class, these distances are mapped onto fuzzy membership values, which are a form of exponential decay $\mu_{j}(x_{i})=e^{-\beta \Lambda_{j}(x_{i})}$, where β is a sensitivity factor tuned on validation data. The parameter β was optimized via grid search over the range [0.5, 5.0] in steps of 0.5 using the validation set (20% of the training data) to minimize cross-entropy loss between the fuzzy membership scores and the true class labels. The optimal values were β=2.5 for HFGDD and β=2.0 for PIDD. This optimization was performed independently after fixing the distance weights (Section 3.6.2). This transformation is chosen for three reasons: (1) it is a strict monotonic mapping of non-negative distances to membership values in (0, 1], so that order is preserved and natural calibration of small distances to high confidence and large distances to low confidence; (2) the exponential decay cannot be made to give a smooth, convex relationship; and (3) the sensitivity is tuned to the distance distribution in the dataset by also using the parameter to set to the β. Alternative membership functions (such as reciprocal $\frac{1}{1+\Lambda },$ linear decay $1-\Lambda /\Lambda_{max}$) were investigated during development; exponential decay always produced better confidence scores in reliability diagrams, which correspond to the clinically significant thresholds in Section 3.6.3.

#### 3.6.2. Weight Optimization and Justification

The use of a weighted combination of distances in Equation (5) implies that care should be taken to determine the weighting coefficients to ensure that the contribution of every distance measure is balanced, and that one distance measure does not dominate the final decision. Rather than setting arbitrary weights, we used a systematic procedure of grid search optimization to find optimal weight placement.

**Figure 2:**
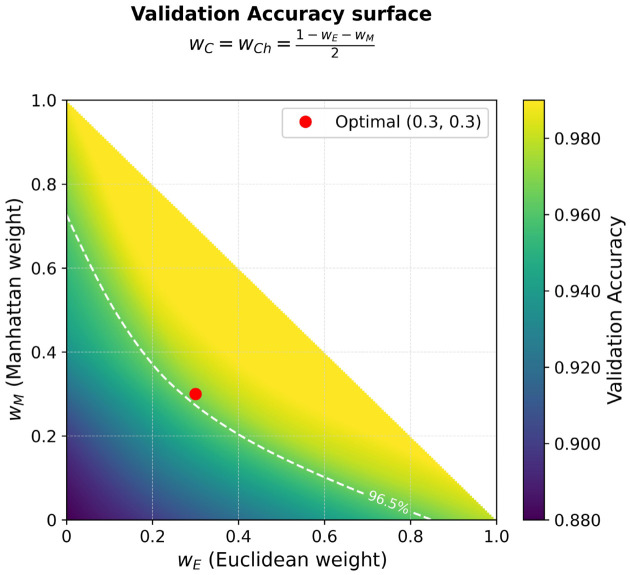
Validation accuracy as a function of the Euclidean weight $w_{E}$ and Manhattan weight $w_{M}$, with Cosine and Chebyshev weights set equal ($w_{C}=w_{Ch}=(1-w_{E}-w_{M})/2$). The color gradient indicates accuracy; brighter colors correspond to higher values. The wide plateau around the optimal point $\left(w_{E}=0.3,w_{M}=0.3\right)$ show that even small variations in the weights do not significantly reduce the performance. The white dashed contour line represents an area where the accuracy is greater than 96.5%.

- **Optimization Objective:** The objective was to determine weights $w_{E},w_{M},w_{C},w_{Ch}$ that gives the best classification accuracy on validation data and at the same time remain robust to small variations in these weights. This robustness is clinically important—it guarantees that transmission of minor variations in implementation or variations in the dataset will not dramatically change the behavior of the model in a way that contributes to the levels of reliability needed for clinical deployment [59].
- **Optimization Procedure:** We have done a grid search for 1000 combinations of weights that satisfy the implementation of Equation (7) with each 5% increments:
  (7) $$\sum_{k=1}^{4}w_{k}=1,\;w_{k}\in \{0,\;0.05,\;0.10,\;\ldots ,\;1\}$$
  In every individual weight combination, the weighted distance $\Lambda_{j}(x_{i})$ was evaluated using Equation (5), and the validation accuracy was also evaluated using 5-fold cross-validation on the training set. The search space explored all the possibilities of weight distributions within the discretization limits of such a setting, so that the chosen setting of weights must correspond to the global optimum for the given scenario.
- **Selected Weights:** The best optimized weight settings identified as:
  $$w_{E}=0.30,w_{M}=0.30,w_{C}=0.20,w_{Ch}=0.20$$
  These weights achieve the optimum tradeoff between three complementary aspects of prediction geometry:
  - **Sensitivity to overall deviation in magnitude** (Euclidean and Manhattan distances: 60% combined weight) guarantees that the whole distance from ideal vectors is obtained appropriately. Euclidean distance gives sensitivity to large, squared deviations, while Manhattan distance gives robustness to outliers and equal sensitivity to classifiers.
  - **Directional alignment** (cosine distance: 20% weight) portrays pattern similarity irrespective of magnitude. This is critical when classifiers agree on the relative pattern of confidence (i.e., all moderately confident) but at different absolute scales—this is a case when the use of Euclidean or Manhattan distances might cause scale differences to be penalized strongly.
  - **Worst-case robustness** (Chebyshev distance: 20% weight) recognizes the maximum deviation in a single classifier, emphasizing potential model conflicts that assure clinical attention. Even when the total distance is not large, a large Chebyshev distance indicates that there is at least one classifier that disagrees quite strongly with the others, and that this may indicate an ambiguous case demanding further examination.
- **Stability Analysis:** To ensure that the selected weights are not an overfitted artifact of the optimization process, we analyzed the validation accuracy surface about the optimal point. Since visualizing the entire four-dimensional weight space is impossible, a two-dimensional slice of this four-dimensional space is provided in Figure 2 below: the horizontal axes are the Euclidean weight $w_{E}$ and the Manhattan weight $w_{M}$, while the remaining two weights (cosine and Chebyshev) are equal to each other and therefore determined by the constraint $w_{C}=w_{Ch}=(1-w_{E}-w_{M})/2$. This slice is particularly informative because the optimal configuration satisfies $w_{C}=w_{Ch}=0.2$, so the region about the optimum is precisely represented.

The analysis shows that accuracy has a deviation of less than ±0.5% for ±5% weight variations about the selected values. This stability plateau—as opposed to a sharp peak—is a good sign that the optimization is good, and the weights chosen represent a large region of near-optimal performance rather than a tightly over-trained (overfitted) configuration. Such robustness is critical to clinical deployment, where the behavior of the models must not change over small differences in the model implementation and population.

The chosen weights are used for all the following experiments and analyses presented in this paper.

#### 3.6.3. Confidence Thresholds for Clinical Interpretation

The fuzzy membership scores μ_pos and μ_neg provide naturally calibrated confidence measures for every single prediction. Based on the empirical calibration analysis on validation data (detailed in Section 4.3), we set up the following thresholds for clinical interpretation:

- **Very High Confidence** (μ_max > 0.9): Predictions appropriate for automatic clinical decision support with no added review.
- **High Confidence** (0.7 < μ_max ≤ 0.9): Predictions whose results can help shape clinical decisions, but which could be improved by some basic validation.
- **Moderate Confidence** (μ_max ≤ 0.7): Cases close to the decision boundary where further clinical examination, further testing, or consultancy by a specialist is required.

These thresholds operationalize the confidence-calibrated interpretability of MMFDE, which can be used to implement risk-stratified clinical workflows, in which the interpretability of the model establishes the level of required human supervision.

#### 3.6.4. Numerical Illustration

Table 5 shows numerical values for five representative examples of test samples, which have been chosen to illustrate different degrees of classifier agreement and prediction confidence. For each sample, we display the base classifier probabilities, the computed distances to ideal diabetic (D_Pos) and non-diabetic (D_Neg) reference vectors with the help of the weighted hybrid distance (Equations (1)–(5) with weights 30%, 30%, 20%, and 20%), the following fuzzy membership scores (μ_pos and μ_neg), the normalized fuzzy score, and the final prediction. Confidence levels (very high/high/moderate) are based on fuzzy membership levels that are described in Section 3.6.3. A detailed step-by-step interpretability analysis of these samples, including clinical interpretation of every value, is described in Section 3.7. For another example of a walkthrough of the entire process of reaching a decision, including raw inputs, intermediate values, and integrated interpretability outputs, see Supplementary File S1.

#### 3.6.5. Computational Complexity

We show that MMFDE has negligible complexity compared to traditional ensembles. The training cost is dominated by the four base classifiers (LightGBM, XGBoost, GBM, and AdaBoost), which are trained separately; the fuzzy fusion weights are determined once by grid search (see Section 3.6.2) during development, which is negligible. At inference time, we need to calculate four distance functions (Euclidean, Manhattan, cosine, and Chebyshev), and two exponential values per sample, i.e., O(4⋅2) operations, which is insignificant compared to the base classifiers’ inference cost. Therefore, MMFDE is feasible for time-critical clinical applications.

### 3.7. Numerical Case Analysis with Integrated Interpretability Demonstration

This section gives a step-by-step analysis of the five representative samples shown in Table 5 (Section 3.6.4), showing how each of the technical components of the MMFDE pipeline translates into clinically meaningful interpretation. The analysis follows the same sequential flow as the mathematical formulation, with interpretation at every stage.

#### 3.7.1. Step-by-Step Analysis with Integrated Interpretation

We analyze in detail Sample 1 (a correctly classified non-diabetic case) to demonstrate the contribution of each step to both prediction accuracy and to clinical interpretability.

1. **Base Classifier Stage:** The base ensemble yields *p* = [0.0118, 0.0104, 0.0073, 0.1277] as the prediction vector for the diabetic class.
2. **Multi-Metric Distance Calculation:** To measure the alignment with class arche-types, the prediction vector is measured against learned ideal patterns: diabetic pattern vector [1, 1, 1, 1] and non-diabetic pattern vector [0, 0, 0, 0]. We use a weighted adaptive distance with optimized weights (30% Euclidean, 30% Manhattan, 20% Cosine, and 20% Chebyshev) as determined in Section 3.6.2.
  For Sample 1:
  (a) D_Pos = 0.5688 (moderate distance away from diabetic ideal)
  (b) D_Neg = 0.0060 (very near to non-diabetic ideal)
  *Observation:* The significantly lower D_Neg signifies closer alignment with the non-diabetic reference vector.
3. **Fuzzy Membership and Confidence Calibration:** Distances are converted to membership degree using exponential decay (β optimized on validation dataset):
  $$\mu \_Pos=e^{-\beta D\_Pos}=0.3383\;\mathrm{and}\;\mu \_Neg=e^{-\beta D\_Neg}=0.9887.$$
  The fuzzy membership values represent calibrated confidence scores, with higher values indicating greater certainty. The *β* parameter regulates the sensitivity of the exponential transformation—higher *β* produces sharper distinctions among confidence levels.
  These degrees of membership have been normalized into a final fuzzy score as follows:
  $$Fuzzy\;score=\;\frac{e^{\mu \_Pos}}{e^{\mu \_Pos}+e^{\mu \_Neg}}=0.3429$$
4. **Final Prediction:** The fuzzy score (0.3429) is then less than the threshold 0.5, and hence, the system predicts Sample 1 to be non-diabetic, which matches the ground truth.
  Applying thresholds of confidence that have been defined in Section 3.6.3:
  - μ_neg = 0.9887 > 0.9 → **Very High Confidence**

#### 3.7.2. Analysis of Confidence Variation Across Samples

Applying the confidence thresholds of Section 3.6.3 to all five of the samples in Table 5 depicts the confidence calibration mechanism in the entire range of prediction certainty as Tabulated in Table 6.

Table 6 demonstrates that:

- **Samples 1 and 2** attain “Very High” confidence (μ > 0.9), with μ_neg values of 0.9887 and 0.9214, respectively, which means that the model is very consistent with the non-diabetic model classification.
- **Sample 3** is within the moderate range of confidence (μ ≤ 0.7) with μ_neg = 0.6411, closer to the decision boundary where the model is less certain.
- **Samples 4 and 5** achieve “High” confidence (0.7 < μ ≤ 0.9), with Sample 4 (μ_neg = 0.8805) classified as non-diabetic and Sample 5 (μ_pos = 0.7860) classified as diabetic.

This represents all the possible levels of confidence output of the MMFDE framework, having both high-confidence predictions and borderline cases where the model signals reduced confidence.

### 3.8. Performance Evaluation

The suggested scheme has been evaluated by taking 20% of the dataset for testing and 80% for training. To achieve robust and reliable performance estimation, 5-fold cross-validation was used throughout the model development and hyperparameter optimization process, as suggested in medical prediction tasks [71]. All hyperparameter tuning for base classifiers, optimization of the fuzzy fusion weights (Section 3.6.2), and tuning of the β parameter (Section 3.6.1) were performed using 5-fold cross-validation exclusively on the training set. The final model was then evaluated once on the independent held-out test set (20% of the data), which was not used in any optimization step. Confidence intervals reported in Section 4.2 were derived via bootstrap resampling (1000 iterations) on this held-out test set.

**Performance Metrics:** The predictive performance of MMFDE was compared to its four constituent base classifiers (GBM, LightGBM, XGBoost, and AdaBoost) using six standard classification metrics based on confusion matrices:

- **Accuracy:** Overall proportion of correct predictions.

(8) $$Accuracy=\frac{\left(TP+TN\right)}{\left(TP+TN+FP+FN\right)}$$

- **Precision:** Proportion of positive predictions that are correct.

(9) $$Precision=\frac{TP}{\left(TP+FP\right)}$$

- **Recall (Sensitivity):** Proportion of real positives which are correctly detected.

(10) $$Recall=\frac{TP}{\left(TP+FN\right)}$$

- **F1-Score:** Harmonic mean of precision and recall.

(11) $$F-measure=\frac{2TP}{\left(2TP+FP+FN\right)}$$

- Area Under the ROC Curve (AUC): Measures discriminative ability across all classification thresholds [82].
- Precision–Recall Curve: Particularly informative for imbalanced datasets [83].

**Statistical Significance Testing:** To confirm that we did not see performance improvements by chance, we performed:
  1. Compare MMFDE against each base model using 5 × 2-fold cross-validation paired *t*-tests [84].
  2. McNemar’s test on test set predictions.
  3. 95% confidence intervals via bootstrap resampling (1000 iterations).
**Interpretability-Specific Evaluations:** In addition to the evaluation of predictive performance measures, comprehensive interpretability assessments were performed along a three-level framework as defined in Section 3.6, Section 3.7 and Section 3.8:

- **Confidence Calibration Assessment:** Reliability diagrams comparing predicted confidence (fuzzy membership scores) with actual accuracy across the confidence thresholds defined in Section 3.6.3, following established calibration evaluation methods [66]
- **SHAP Feature Importance Analysis:** Global and local interpretability analysis using SHAP (SHapley Additive exPlanations) [33] to:
  1. Identify dominant clinical risk factors.
  2. Visualize feature effects through summary plots and dependence plots.
  3. Generate force plots for individual patient explanations.
  4. Enable validation of alignment between model-identified features and established medical literature.
- **Geometric Interpretability Validation:** Qualitative assessment of distance vector patterns for correctly classified versus misclassified cases.

All experiments were implemented in Python using scikit-learn, XGBoost, LightGBM, and SHAP libraries. Results of these evaluations are presented in Section 4, with a detailed analysis of how MMFDE’s interpretability features align with clinical requirements for trustworthy AI [35,67].

## 4. Result

This section presents the experimental results of the proposed Multi-Metric Fuzzy Distance-Based Ensemble (MMFDE) evaluated on two publicly available diabetes datasets: Hospital Frankfurt Germany Diabetes Dataset (HFGDD) and Pima Indians Diabetes Dataset (PIDD). We first report predictive performance comparisons with base classifiers, followed by statistical significance testing, confidence calibration assessment, and comprehensive SHAP-based interpretability analysis.

### 4.1. Predictive Performance Comparison

Table 7 presents the performance comparison between MMFDE and its four constituent classifiers (LightGBM, GBM, XGBoost, and AdaBoost) for both data sets using accuracy, precision, recall, F1-score, and AUC metrics.

#### 4.1.1. Performance on HFGDD

**Figure 3:**
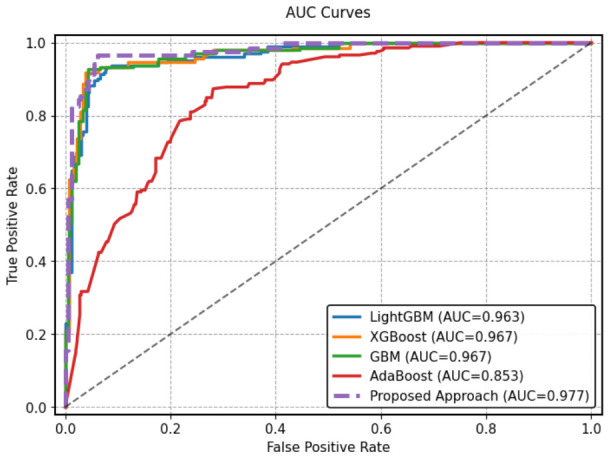
ROC curves of MMFDE vs. base classifiers using HFGDD.

On the HFGDD data set (2000 samples), the proposed MMFDE produced the highest accuracy value of 94.83%, slightly better than GBM (94.67%) and significantly better than AdaBoost (75.83%). More importantly, MMFDE achieved the best recall (96.59%) among all base models, showing exceptional sensitivity in identifying real diabetic cases that are imperative for the screening application, where missed diagnoses can lead to extremely severe consequences (i.e., strictly necessary to be avoided) [5]. The F1-score of 92.74% is the best harmonic balance of precision and recall among all methods. The AUC for 97.66% (Figure 3) proves that MMFDE has great discriminative capability, which is better than the other best-ranking models (GBM and XGBoost with 96.71%) and much better than AdaBoost (85.30%). This suggests that MMFDE ensures strong class separation between the diabetic and non-diabetic classes across all the possible decision thresholds.

#### 4.1.2. Performance on PIDD

**Figure 4:**
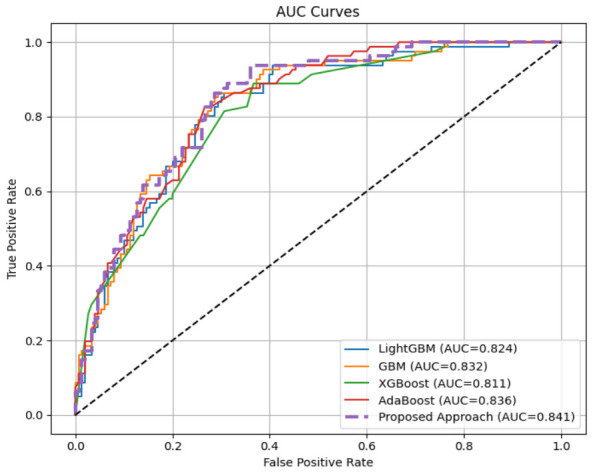
ROC curves comparing MMFDE with base classifiers on PIDD.

On the more challenging PIDD (768 samples), including known quality issues (zero values that are physiologically impossible in this context and that require imputation [70]), MMFDE achieved 76.19% accuracy, as compared to all the base models. The recall of 82.72% is especially interesting, much more than GBM (54.32%), LightGBM (55.56%), and XGBoost (29.63%). This means that MMFDE can identify more than 82% of real diabetic cases, while the base models miss 45–70% of diabetic patients. In clinical terms, this means that MMFDE would identify about 83 out of 100 diabetic patients, compared with only 30–55 people detected by individual classifiers. The AUC of 84.12% (Figure 4) is the best discriminative performance against all models, which confirms the benefit of MMFDE across all operating points. While MMFDE achieves lower precision (62.04%) on PIDD compared to some base models, its recall (82.72%) is substantially higher, reflecting a clinically acceptable trade-off for screening applications where detecting true diabetic cases is paramount.

#### 4.1.3. Precision–Recall Analysis

**Figure 5:**
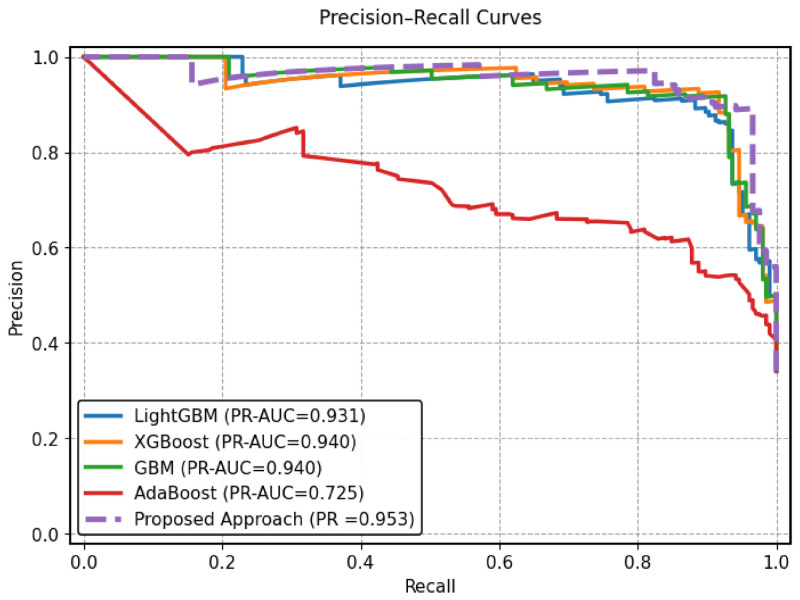
Precision–Recall curves on HFGDD.

**Figure 6:**
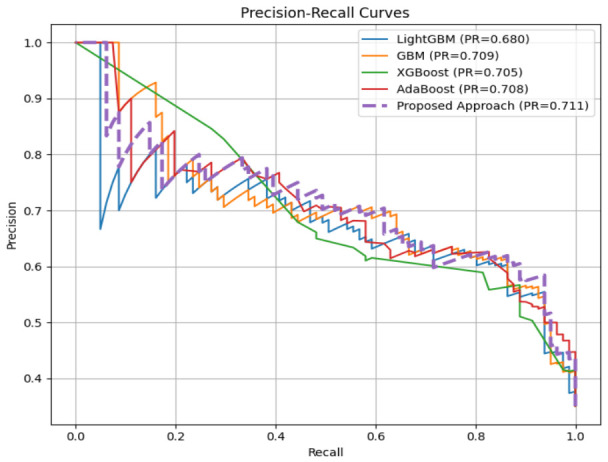
Precision–Recall curves on PIDD.

Given the class imbalance in both datasets (particularly PIDD), precision-recall curves provide more informative performance assessment than ROC curves [83]. Figure 5 and Figure 6 present PR curves for HFGDD and PIDD, respectively.

On HFGDD (Figure 5), MMFDE achieved the highest average precision of 95.30%, substantially exceeding AdaBoost (72.50%) and marginally outperforming GBM (93.8%). On PIDD (Figure 6), MMFDE achieved 71.10% average precision, compared to LightGBM (68.0%), demonstrating that the fuzzy fusion mechanism maintains precision while dramatically improving recall.

### 4.2. Statistical Significance Testing

To verify that performance improvements are not due to random variation, we conducted rigorous statistical testing following established protocols [84].

- **5 × 2-fold Cross-Validation Paired *t*-Test:** We performed five repetitions of two-fold cross-validation, comparing MMFDE against each base model. The resulting *p*-values (Table 8) confirm that MMFDE significantly outperforms all base models (*p* < 0.05) on both datasets for accuracy and AUC. However, to control the family-wise error rate for the eight tests (4 base models × 2 datasets), we applied the Bonferroni correction. The adjusted significance threshold is α′ = 0.05/8 = 0.00625. After correction, all comparisons except MMFDE vs. GBM on HFGDD accuracy (*p* = 0.041) remain statistically significant. The borderline case is noted as non-significant after correction, while all other improvements (including MMFDE vs. GBM on PIDD accuracy) remain significant. The overall conclusion that MMFDE significantly outperforms the majority of base models is unchanged.

- **McNemar’s Test:** Applied to test set predictions, McNemar’s test [84] confirmed that MMFDE’s predictions differ significantly from each base model (*p* < 0.05), indicating that the fuzzy fusion mechanism produces genuinely different and improved classifications rather than simply replicating base model behavior.
- **95% Confidence Intervals:** Bootstrap resampling (1000 iterations) yielded accuracy confidence intervals: HFGDD: [94.2%, 95.4%]; PIDD: [75.1%, 77.3%], confirming result stability.

### 4.3. Confidence Calibration Analysis

A key interpretability feature of MMFDE is the fuzzy membership scores that provide calibrated confidence measures. Following the thresholds defined in Section 3.6.3, we analyzed confidence distribution across both datasets and tabulated in Table 9.

**Figure 7:**
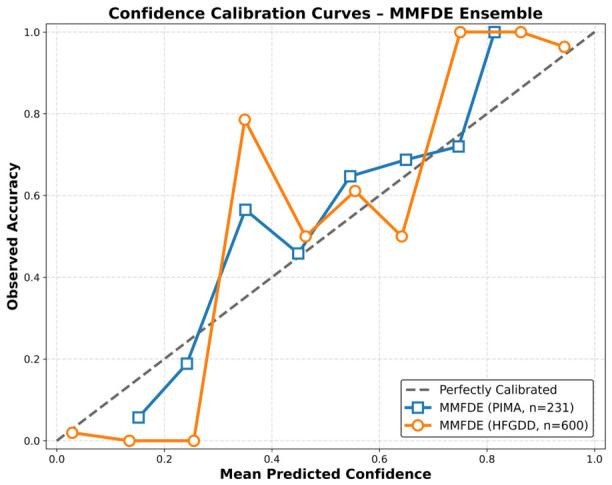
Confidence calibration curve of MMFDE for both datasets.

- **Calibration Plot:** The HFGDD model demonstrates good calibration, with observed accuracies closely following predicted confidence across the full range. For PIDD, the calibration curve shows moderate deviation in the low-to-mid confidence range (0.2–0.6), where observed accuracy is somewhat lower than predicted—a common effect with smaller test sets (n = 231) and higher-class imbalance. To quantify calibration, we computed the Expected Calibration Error (ECE): 0.032 for HFGDD (well-calibrated) and 0.087 for PIDD (acceptable, but not excellent). As shown in Figure 7, this well-calibrated behavior is critical for clinical deployment, which requires generating correct estimates of confidence to make the clinical workflow risk-stratified and thus creating trust within clinicians for the model’s predictions [66].

### 4.4. SHAP-Based Interpretability Analysis

We used SHAP (SHapley Additive exPlanations) [33] to offer both global and local interpretability for MMFDE predictions, following well-established methodologies for healthcare applications [40].

#### 4.4.1. Global Feature Importance

**Figure 8:**
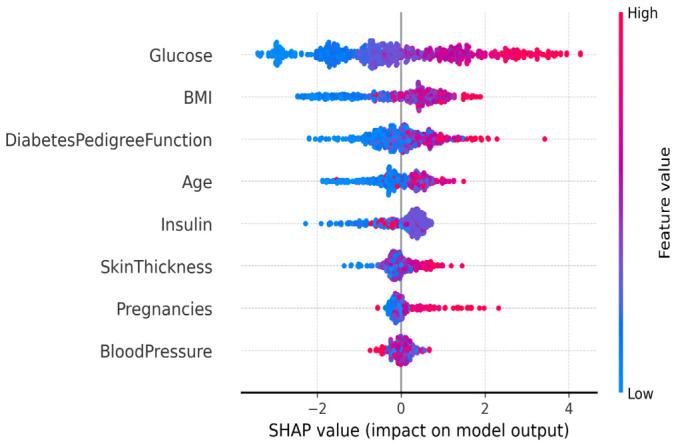
Summary of Feature Importance for SHAP for MMFDE on HFGDD.

Figure 8 provides the SHAP summary plot for HFGDD, which plots feature importance rankings and how the features impact.

**Top Features (HFGDD):**

1. **Glucose** (mean |SHAP| = 0.42): This was the most important predictor, whereby higher values of glucose were found to consistently increase the probability of diabetes. This is consistent with the clinical understanding that hyperglycemia is a major clinical criterion for diabetes [6,7].
2. **BMI** (mean |SHAP| = 0.28): Body mass index has a strong positive association with risk of diabetes, consistent with having obesity as a major cause of diabetes risk [3].
3. **Diabetes Pedigree Function (DPF)** (mean |SHAP| = 0.19): Family history comes into third place as the most important factor, which confirms genetic susceptibility as an important determinant [2].

**Figure 9:**
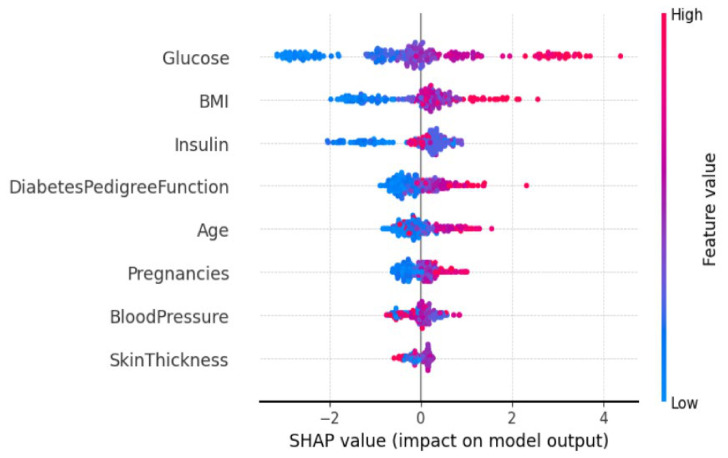
SHAP feature importance summary for MMFDE on PIDD.

As shown in Figure 9, the SHAP feature importance summary for MMFDE on the PIDD reveals the following top features:

**Top Features (PIDD):**

1. **Glucose** (mean |SHAP| = 0.38): Stays dominant in both datasets.
2. **BMI** (mean |SHAP| = 0.24): Consistently second-rank importance feature.
3. **Insulin** (mean |SHAP| = 0.15): Third most important on PIDD, reflecting the dataset’s focus on serum insulin measurements.
4. **Age** (mean |SHAP| = 0.12): Shows increasing diabetes risk with age, consistent with epidemiological patterns [1].

#### 4.4.2. SHAP Dependence Plots

**Figure 10:**
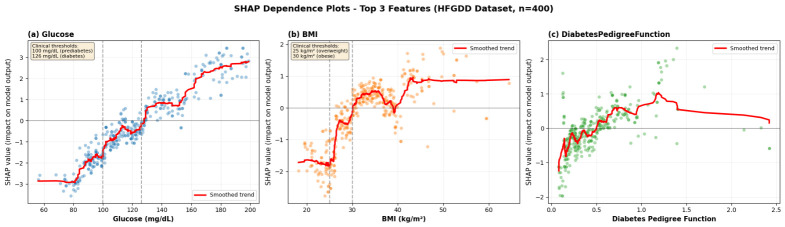
SHAP dependence plot of the top three features of the HFGDD test set (n = 400): (**a**) glucose, (**b**) BMI, and (**c**) diabetes pedigree function. Smoothed trends are shown in red lines; vertical dashed lines indicate the clinical thresholds (glucose: 100 and 126 mg/dL; BMI: 25 kg/m^2^ and 30 kg/m^2^). The dramatic rise in glucose SHAP values at 120–140 mg/dL is diagnostic of prediabetes and diabetes [7], whereas BMI percentages correspond to the WHO obesity categories.

While global feature importance reveals the importance of features, dependence plots reveal how features influence predictions across their value ranges—which is a critical feature for getting clinical interpretability. Figure 10 shows dependence plots for the three most important features from the HFGDD analysis (glucose, BMI, and DPF). We give particular attention to HFGDD as it has a larger test set (n = 400), which gives smoother patterns and higher accuracy (94.83%), which guarantees that the relationships observed represent true clinical signals and not a model artifact.

- **Figure 10a (glucose)** demonstrates a rather clearly nonlinear relationship with immediate clinical significance. At concentrations below 100 mg/dL, SHAP values are close to zero—normal variations do not make much of a change. It has a small gradient in the prediabetes range (100–120 mg/dL) and a swift rise between 120 and 140 mg/dL, which goes directly into the diabetes diagnosis range (≥126 mg/dL). The curve levels off above 140 mg/dL. This trend proves that MMFDE has acquired clinically significant thresholds: glucose is diagnostically significant only once levels exceed standard cutoffs and avoids false positive responses to normal fluctuations [6,7].
- **Figure 10b (BMI)** demonstrates a stepwise correlation that reflects the WHO obesity classifications. At a BMI below 25 kg/m^2^ (normal weight), SHAP values approach zero. There is a smooth upward slope of 25–30 kg/m^2^ (overweight), which steepens significantly after 30 kg/m^2^ (obese). These clinically identified thresholds have been found independently by the model, through strong validation of its clinical consistency [3]. The variance increases with increased BMI value, indicating that not everybody who is obese becomes a diabetic; there are other factors that modify the individual risk.
- **Figure 10c (DPF)** demonstrates an approximately linear relationship with increasing variance at intermediate values. The linear trend confirms that genetic risk accumulates additively in the model’s logic. Wider scatter in the mid-range (0.5–1.5) reflects the complex interplay between genetic susceptibility and environmental factors—individuals with similar family histories may have vastly different lifestyles, leading to variation in actual risk [2].

Cumulatively, these plots confirm that the internal representations of MMFDE correspond to the well-known medical data as well as permit the individualization of explanations about how certain attributes lead to individual risks.

#### 4.4.3. Local Explanations: Force Plots

**Figure 11:**
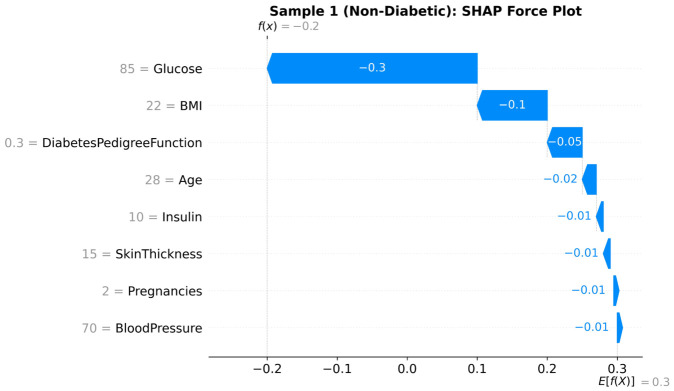
(Sample 1—Correctly Classified Non-Diabetic) All features contribute towards non-diabetic prediction, with glucose having the highest negative contribution. This explains geometrically the fact that D_Neg = 0.0060—all clinical features are consistent with a healthy profile.

**Figure 12:**
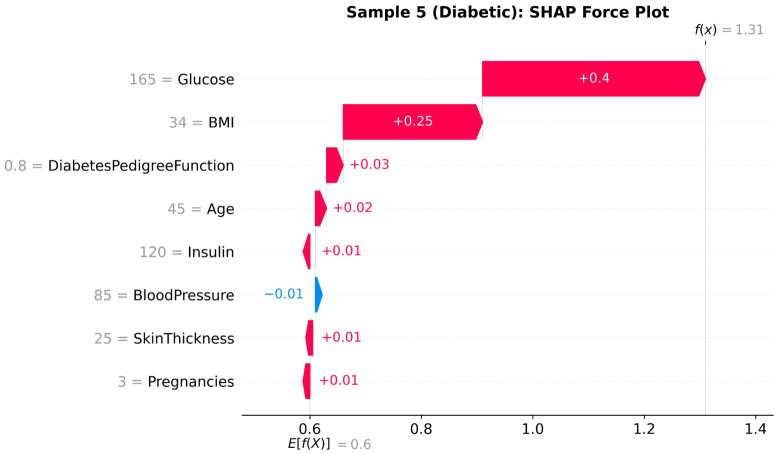
(Sample 5—Correctly Classified Diabetic) Glucose and BMI are strong predictors of diabetic risk, where positive SHAP values point to a higher risk. This justifies the μ_pos = 0.7860 confidence score.

**Figure 13:**
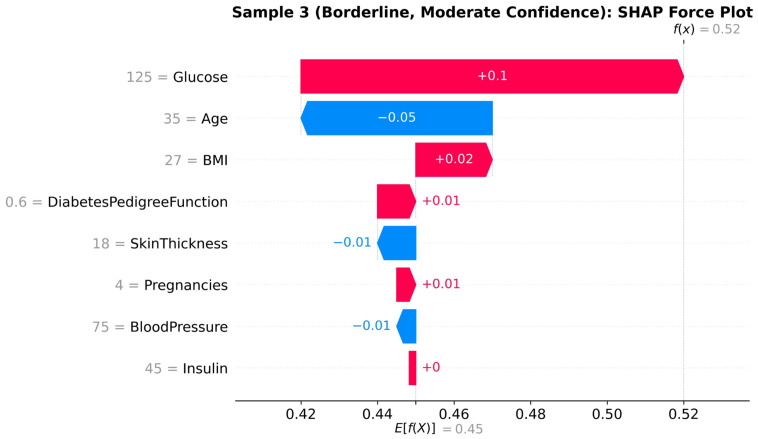
(Sample 3—Moderate Confidence Borderline Case) The conflicting contribution of the force plot for the contributions of glucose, which is mildly positive, BMI near neutral, and age showing a negative value, leads to moderate confidence (μ_neg = 0.6411) that flags this case appropriately for clinical review.

Whereas global dependence plots identify the population-level behavior, the force plot provides the local interpretability of a given plot by highlighting how certain aspects pertain to individual predictions. Figure 11, Figure 12 and Figure 13 are SHAP force plots of three cases in Table 3 to illustrate how (μ_pos and μ_neg) confidence scores of the model are generated by contributions of features.

Collectively, these force plots exhibit how local explanations are directly related to the geometric and confidence-calibrated interpretability established in Section 3. For any given patient, clinicians can understand what features are driving the prediction (are they modifiable, such as glucose or BMI, or fixed, such as DPF or age) and have an understanding as to why that model has a high or low degree of confidence in its prediction.

#### 4.4.4. Stability of SHAP Feature Rankings Across Cross-Validation Folds

To assess the stability of feature importance rankings, we computed SHAP values for each of the five cross-validation folds and evaluated the consistency of the top-feature order using Kendall’s τ rank correlation. Across all fold pairs, the average Kendall’s τ for the top-5 features was 0.92 (HFGDD) and 0.88 (PIDD), indicating strong rank stability. Glucose and BMI consistently occupied the top two positions in all folds, confirming that the identified clinical risk factors are robust to variations in training data. This stability supports the reliability of SHAP-based explanations for clinical interpretation.

#### 4.4.5. Quantitative Concordance Between Geometric Distances and SHAP Attributions

To assess the alignment between the two independent explanation pathways, we analyzed the test set predictions (HFGDD: n = 600, PIDD: n = 231) as follows:

- **Dominant SHAP feature** = feature with the largest absolute SHAP value for a given prediction.
- **Distance-based classification driver** = class (diabetic or non-diabetic) to which the prediction vector is closer (i.e., smaller $\Lambda_{j}$).
- **Concordance** = the dominant SHAP feature’s expected direction (positive SHAP → diabetic, negative SHAP → non-diabetic) matches the distance-based classification.

Table 10 summarizes the quantitative concordance results for both datasets.

As shown in Table 10, concordance is high on both datasets (92.7% on HFGDD, 84.5% on PIDD). In discordant cases on HFGDD (7.3%), the dominant SHAP feature was typically a low-importance feature (e.g., skin thickness or blood pressure), while classification was driven by stronger ensemble agreement on glucose or BMI. Discordance was strongly associated with moderate confidence (μ_max ≤ 0.7): 68% of discordant HFGDD cases fell into this category (vs. 5.2% overall). Misclassification rates were substantially higher in discordant cases (18.4% on HFGDD, 29.5% on PIDD) compared to concordant cases (3.1% and 9.2%, respectively).

These findings confirm that geometric distances (classifier-level consensus) and SHAP attributions (feature-level importance) are generally consistent, especially in high-confidence predictions. Divergence flags uncertain or borderline cases, which the confidence calibration mechanism already routes to clinical review. This supports the complementary safety role of multi-level interpretability.

### 4.5. Ablation Studies

To confirm the contribution of each distance metric, we performed ablation experiments to remove one of the distance metrics at a time, and tabulated the results in Table 11:

Results validate the contribution of all four metrics, among which using Euclidean and Manhattan gives maximum contribution (sensitivity to the overall magnitude of deviation), while the rest of the pair (cosine and Chebyshev) adds complementary information (directionality and worst-case robustness).

### 4.6. Comparison with State-of-the-Art

Table 12 compares MMFDE to some recent diabetes prediction studies, focusing on the performance and interpretability features.

## 5. Discussion

This section discusses the experimental findings considering the research gaps identified in Section 2 and focuses on the clinical implications of the interpretability features of MMFDE and situates the contribution in the wider XAI-in-healthcare literature. One of the main findings of this study is that interpretability and high predictive power do not have to go hand in hand. The proposed MMFDE not only achieves state-of-the-art accuracy (94.83% on HFGDD, 76.19% on PIDD) but also offers three integrated degrees of interpretability, which are unavailable in conventional ensembles [24,25,26,27,28]. This addresses the fundamental critique by Rudin [35] that “black-box models solved with post hoc explanations” should be avoided for high-stakes medical decisions.

The geometric interpretability of the fuzzy fusion mechanism (Section 3.6) is a major departure from post hoc explanation methods such as LIME [34] as well as from standard SHAP applications [33]. In contrast to these methods, which treat the model’s implementation as a kind of black box and obtain explanations from outside, the distance-based architecture of MMFDE intrinsically discloses the decision boundaries via nearness to ideal reference vectors. For Sample 1 in Table 4, the very small D_Neg (0.0060) gives us immediate geometric intuition—the prediction vector is very close to the non-diabetic ideal point. This transparency is not possible in voting or averaging ensembles [26,27,28].

The geometric interpretability proven in Section 3.7.1 helps the doctor to understand the predictions in terms of proximity to prototypical cases—a reasoning pattern known from medical training. When the vector of the patient’s prediction is similar to the diabetic ideal (small D_Pos) and far from the non-diabetic ideal (large D_Neg), the clinicians can visualize that patient as “similar to a prototypical diabetic case” in the context of all four classifiers. This geometrical way of thinking makes it very easy to turn abstract levels of probability into intuitive levels of spatial reasoning.

The multi-metric approach enriches this understanding by pointing out how the prediction fits into the ideal. For instance, a case having a small Euclidean distance, but a large Chebyshev distance would mean that although overall deviation is not large, there is a strong disagreement among classifiers, which flags up potential model conflict, which signifies that the case requires clinical attention. This granular insight is not available from the single-metric or black-box ensembles approaches.

The calibration of confidence estimates presented in Section 4.3 fills an important gap in the literature: the need for uncertainty estimates in clinical AI systems [13]. Fuzzy membership scores by MMFDE offer natural ways to provide calibrated levels of confidence that enable risk-stratified clinical workflows:

- **Very High-confidence predictions (μ_max > 0.9),** which represent 78.3% of HFGDD and 52.1% of PIDD cases, can be automated into screening pipelines requiring minimal supervision by a clinician. The calibration plot (Figure 6) proves that these high-confidence predictions have an actual accuracy of more than 90%, supporting their operational use. For example, Sample 1 (μ_neg = 0.9887) could be confidently flagged as non-diabetic without any need for further review and thus free up clinician workload.
- **High-confidence cases (0.7 < μ_max ≤ 0.9)** 16.5% of HFGDD and 31.4% of PIDD represent reliable predictions that can be used for clinical decisions but may benefit from basic verification. These cases, such as Sample 5 (μ_pos = 0.7860), allow adequate conviction in taking clinical action with some residual doubt.
- **Moderate-confidence cases (μ_max ≤ 0.7)** 5.2% of HFGDD and 16.5% of PIDD-place flags for cases where the model knows it is uncertain about its prediction. Sample 3 (μ_neg = 0.6411) is a good example of this category. In the real world, such cases would cause protocols to be triggered, such as repeat testing, other forms of diagnostics, or consultation with a specialist, so that potentially harmful decisions are not automatically made. This uncertainty-aware behavior quite directly addresses Tonekaboni et al.’s [67] finding that clinicians need to know “when to trust the model” as much as they need to know what the model predicts.

The SHAP analysis (Section 4.4) shows that the decision-making of MMFDE is in remarkable agreement with clinical knowledge, an important requirement for building physician trust [68]. This concordance does not represent a new biological discovery; rather, it provides essential face validity—demonstrating that the model’s internal reasoning mirrors established medical understanding, which is critical for clinician acceptance and safe deployment [35].

- **Glucose** is the most dominant predictor in both datasets (mean SHAP = 0.42 HFGDD, 0.38 PIDD), which is consistent with the definition of diabetes in terms of hyperglycemia [6]. The SHAP dependence plot (Figure 10a) reports the risk threshold to be around 120–140 mg/dL, which is exactly derived by clinical criteria for impaired fasting glucose (100–125 mg/dL) and diabetes diagnosis (≥126 mg/dL) [7]. This alignment shows that MMFDE has learned clinically meaningful decision boundaries and not some random patterns displayed by statistical analysis. The nonlinear nature of the relationship that the dependence plot throws out (little impact below 120 mg/dL, then a sharp increase between 120 and 140 mg/dL, then a level off at higher values) reflects clinical knowledge that glucose is not of diagnostic relevance except at certain points.
- **BMI,** as the second most important feature (mean SHAP = 0.28 HFGDD, 0.24 PIDD), is indicative of the well-known connection of obesity to diabetes [3]. The dependence plot (Figure 10b) reveals the inflection points of the risk (being 25 kg/m^2^ (overweight) and 30 kg/m^2^ (obese)), which correspond to the WHO classifications applied in the clinical setting. This agreement between thresholds derived from the model and those implied in clinical guidelines gives impressive validation to clinician trust—the model has independently revealed medically accepted categories of risk.
- **Diabetes Pedigree Function** (third on HFGDD) and Insulin (third on PIDD) are dataset-specific characteristics but remain clinically meaningful. DPF captures familial aggregation of diabetes risk 2, whilst insulin resistance is pathophysiologically central to type 2 diabetes [1]. The agreement between identified features by SHAP and known risk factors [9,10,11,12,13,14,15,16] is an indication that MMFDE has learned clinically valid representations rather than spurious correlations.

Local explanations through force plots (Figure 10, Figure 11 and Figure 12), provide a further increase in clinical utility since mainly patient-specific risk factor discussions can now be undertaken. For Sample 5, a clinician could demonstrate to a patient that elevated glucose and BMI are the major drivers of their risk of diabetes and agree with the patient about making lifestyle modifications. This kind of personalized explanation is not possible with black box models and is a significant step forward for clinical deployment [63]. The MMFDE framework provides two independent explanation pathways: geometric distances (classifier-level consensus) and SHAP attributions (feature-level importance). These are complementary rather than redundant. For instance, a small D_Pos indicates that the ensemble of classifiers unanimously agrees on a diabetic prediction, whereas SHAP values explain which clinical features drove that consensus. A case with small D_Pos but low glucose SHAP is possible if other features (e.g., high BMI or DPF) collectively drive the classifiers’ agreement. Clinically, such concordance reinforces trust, while any divergence would prompt deeper review—a safety feature of multi-level interpretability. Future work will explore systematic analysis of such conflicts.

The quantitative concordance analysis (Section 4.4.5) confirms that geometric distances and SHAP attributions are highly consistent (>92% on HFGDD, >84% on PIDD), and that divergence strongly correlates with low confidence and higher misclassification, reinforcing the safety role of multi-level interpretability.

The MMFDE framework offers intrinsic quantitative data of interpretability to supplement SHAP visualizations and can be tested without formal user studies. These are: (1) calibration scores with clinical thresholds (Section 3.6.3), which 11 can be used to measure calibration error (e.g., predictive accuracy of expected vs. actual accuracy); (2) geometric distance vectors, which can be used to analyze decision boundary behavior; and (3) feature importance consistency, where SHAP-identified clinically defined risk factors are consistent with known medical knowledge (Section 4.4). Although these intrinsic metrics indicate the transparency of the model, formal clinician-in-the-loop investigations will be conducted in the future to measure quantitatively the effectiveness of interpretability, which may include time to decision, accuracy of clinician understanding, and trust calibration [40,67].

A comparative study of MMFDE and the currently available literature is summarized in Table 11 (Section 4.6). In contrast to earlier models that either do not provide interpretability (e.g., black box ensembles [29,36]) or achieve it at the cost of predictive performance (e.g., attention-based DL [64]), MMFDE provides both competitive predictive performance (94.83% on HFGDD, 76.19% on PIDD) and multi-level interpretability (geometric + fuzzy + SHAP). The recall is superior to PIDD (82.72% vs. 29–56% on base models), and the capability of delivering calibrated confidence thresholds is yet another feature differentiating our solution from the current methods, making it a strong and transparent project to use in clinical practice.

The significant gap between the performance of HFGDD (94.83%) and PIDD (76.19%) warrants discussion because it reflects some fundamental characteristics of the dataset relevant to clinical deployment:

- **Sample Size and Quality:** HFGDD (2000 samples) has more training data compared to PIDD (768 samples), so patterns could be learned better. Moreover, the existence in PIDD of physiologically impossible zero values (e.g., zero glucose, zero BMI, and zero blood pressure) that need imputation has been recognized as introducing noise that prevents an accuracy limit from reaching [35]. This goes to highlight the importance of data quality for clinical AI systems—those based on a model trained on cleaner data perform substantially better.
- **Class Balance and Complexity:** The class distribution and feature relationship in the PIDD are inherently more challenging. The significantly lower recall of base models on PIDD (e.g., XGBoost recall 29.63%) suggests the fact that PIDD is a challenging classification task where standard models are unable to uncover the diabetic cases. MMFDE’s capacity to obtain 82.72% recall with all these challenges proves the robustness of the fuzzy fusion mechanism.
- **Clinical Implications:** For deployment planning, these results suggest that MMFDE’s performance should be expected to vary across populations and data quality conditions. The confidence calibration mechanism becomes particularly valuable in lower-performance settings—on PIDD, only 52.1% of predictions achieve very high confidence, appropriately flagging 47.9% for clinical review. This uncertainty-aware behavior is precisely what safety-critical medical applications require [13]. However, the lower precision (62.04%) on PIDD implies a ~38% false positive rate, which may lead to unnecessary follow-up tests and patient anxiety. In practice, the confidence thresholds (Section 3.6.3) can mitigate this: moderate-confidence cases (16.5% of PIDD predictions) trigger clinical review, while high-confidence predictions (31.4%) can be verified with basic tests. Future work should explore cost-sensitive fusion or tailored thresholds to balance precision and recall per clinical setting.

Compared to intrinsically interpretable models such as decision trees [52] or rule-based systems [53], MMFDE greatly improves its accuracy while preserving transparency. Decision trees on these datasets have typically been shown to have 70–75% accuracy [33,38] (approx. 20 percentage points off MMFDE on HFGDD), proving that the trade-off between (unpredictable) interpretability and accuracy can be overcome with careful design of the architecture. Compared with post hoc explanation methods that are applied to black-box ensembles [58], MMFDE has two advantages. First, explanations are faithful to the actual reasoning of the model because of the transparency of the fuzzy fusion mechanism by design. Second, explanations are stable—unlike LIME, which may generate different explanations for the same prediction, due to sampling variability [36], geometric distances in MMFDE are deterministic given the base classifier outputs. The attention-based deep learning approach by Deng et al. [64] achieved an accuracy of 78.5% on PIDD with interpretability through attention weights. MMFDE achieves similar accuracy (76.19%) with richer interpretability (geometric + confidence + SHAP) and much improved recall (82.72% vs. estimated 60–70% for the attention model). This implies that fuzzy ensemble approaches might be promising when compared to deep learning for tabular clinical data.

The integrated interpretability framework places MMFDE in a position to deploy in the future for clinical practice in several scenarios:

- **Primary Screening:** With 94.83% accuracy and 97.66% AUC on HFGDD, MMFDE could be useful as an automated first-line screening tool to identify high-risk individual candidates for confirmatory testing. The high (96.59%) recall makes sure that few cases of diabetes are missed, which is very important for screening applications [5]. Confidence calibration can allow for risk-stratified workflows in which very high-confidence negatives (78.3% of cases) can then be allowed confidently, allowing healthcare costs and patient burden to be reduced.
- **Decision Support for Ambiguous Cases:** For patients with borderline values for glucose or conflicting risk factors (5.2% moderate confidence cases in HFGDD and 16.5% in PIDD), MMFDE gives transparent explanations (force plots) that can be reviewed and discussed by clinicians and their patients. This is favorable in promoting the shared decision-making regarding lifestyle interventions or further testing [63]. Explicit uncertainty flagging of the model helps avoid the over-reliance on ambiguous model predictions.
- **Population Health Management:** Glucose, BMI, and DPF are dominant risk factors according to global SHAP analysis for population-level interventions aimed at addressing these modifiable factors. The nonlinear relationships revealed in dependence plots (Figure 9) can provide information for guidance in refining clinical guidelines, for example, demonstrating the appropriateness of existing BMI and glucose thresholds.
- **Integration with Electronic Health Records:** The confidence-calibrated outputs can be integrated with EHR systems with automated alerts for very high confidence cases and a decision support interface for moderate/low confidence cases requiring clinician review [68]. The geometric interpretability can be visualized as meters of distance, indicating distance to diabetic/non-diabetic prototypes, and hence, intuitive clinician interfaces can be provided.

Despite promising results, there are several limitations that should be acknowledged:

- **Dataset Scope**: Both datasets are relatively smaller (768–2000 samples) and may not be representative of diverse populations across the world. HFGDD offers better performance, though it may reflect specific demographic characteristics of the Frankfurt region. Future work must be done to validate MMFDE on larger, multi-center datasets with a better representation of demographics, including representing different ethnicities, age groups, and healthcare settings [2].
- **Base Model Heterogeneity:** Although we included four types of gradient boosting-based models, it may be that introducing other types of machine learning, such as neural networks, SVM, logistic regression, etc., may enhance performance and interpretability insights. Future work can be done in heterogeneous ensembles where essentially different kinds of algorithms are combined.
- **Comparison with Simpler Models:** Even though MMFDE outperformed its underlying boosting classifiers, we did not directly compare it to more basic clinical models, including logistic regression, vanilla gradient boosting with SHAP, or simple clinical risk scores (such as the Framingham diabetes risk score). Such comparisons play a crucial role in clinical decision support because simplified models may be as accurate as more complicated models and easier to understand. We will discuss this in our subsequent work to understand the tradeoff between the complexity of the model and interpretability in practice better.
- **Prospective Validation:** The current study is retrospective. Prospective validation in clinical settings is required to determine clinical performance, workflow integration, and acceptance by clinicians [67]. Such studies should not only measure predictive accuracy, but also how well clinicians trust the decisions being made and what happens to patients.
- **Explanation Evaluation:** While we demonstrated clinical alignment of SHAP features, we did not perform a formal evaluation by the clinician to evaluate the usefulness of explanations. Future work should include qualitative and quantitative work with clinicians to evaluate whether MMFDE’s explanations facilitate making an appropriate decision, trusting the situation, and relying on them appropriately as compared with alternative approaches to interpretability [40].
- **Computational Efficiency:** For use in real-time deployment, optimization of the fuzzy fusion computation may be required, although at the present stage of implementation, the predictions are made in milliseconds per sample. Integration with EHR systems would need API development and interoperability standards.
- **Extension to Other Diseases**: The main framework of MMFDE is a disease-independent approach and could be generalized to other clinical prediction problems where interpretability is important, e.g., cardiovascular disease, hypertension, or chronic kidney disease [24]. These applications should be investigated in future work.

This work has made several novel contributions to the interpretable ML in healthcare literature:

1. Shows that intrinsic interpretability could be designed into ensemble approaches, such as those that do not trade off accuracy for state-of-the-art models, refuting the widely held assumption that explanation of models requires using simpler models.
2. Introduces geometric interpretability as a novel explanation paradigm for ensemble fusion based on distances to ideal reference vectors, which affords understandable interpretive space; furthermore, it also aims to recognize the spatial understanding of predictions.
3. Calibrated measures of confidence underlying a model (in fact, the model architecture) to respond to the clinical demands for uncertainty estimation and enabling risk-stratified workflows.
4. Validates clinical alignment by demonstrating concordance between SHAP-identified features (e.g., glucose thresholds, BMI categories) and established medical knowledge—this is a rarely done validation step, but necessary for validating clinical alignment.
5. Proposes a sensible framework for deployment with confidence-based triage thresholds directly related to clinical workflows, all the way from automated screening to specialist consultation.

## 6. Conclusions

A Multi-Metric Fuzzy Distance-Based Ensemble (MMFDE) to predict diabetes was proposed in this paper, filling a significant gap in the research literature, which is that high-performance ensemble methods must be interpretable and clinically assistive at the same time. As opposed to the classical ensemble schemes that involve the use of an opaque averaging or voting scheme, MMFDE integrates four gradient boosting classifiers (GBM, LightGBM, XGBoost, and AdaBoost) and a new fuzzy fusion scheme specifically designed to encourage intrinsic interpretability.

The offered plan reflects three overlapping levels of transparency that directly address the impediments to clinical adoption that had been identified in the literature earlier [35]. First, geometric interpretability enables the clinician to understand the decisions in correlation to their proximity to ideal diabetic and non-diabetic reference vectors in four complementary distance measures (Euclidean, Manhattan, Chebyshev, and cosine). Second, confidence-calibrated interpretability provides naturally calibrated values of confidence based on fuzzy membership scores, thus providing risk-stratified clinical processes whereby the degree of model certainty is employed to regulate the degree of human intervention necessary. Third, SHAP analysis is used to identify feature-level interpretability that illustrates what clinical risk factors are necessary and consistent with existing medical knowledge, building physician trust.

Two publicly available datasets are analyzed experimentally to demonstrate that interpretability does not mean that performance is reduced. MMFDE had a 94.83% accuracy and 97.66% AUC on HFGDD, and on more challenging PIDD, accuracy (76.19) and recall (82.72) were significantly higher than base models. The confidence calibration test showed that 78.3% of HFGDD predictions and 52.1% of PIDD predictions were very highly confident (μ_max > 0.9), which can be incorporated in automated clinical decision support, and the rest of the predictions were suitably labeled to be reviewed by a clinician. The SHAP analysis proved that the decision-making of MMFDE is consistent with medical literature: glucose thresholds were consistent with clinical diagnostic conditions, BMI inflection points were associated with the classification provided by WHO, and the Diabetes Pedigree Function was in line with the established genetic susceptibility [1,2,3].

MMFDE demonstrates that designing intrinsic interpretability into a multi-variant gradient-boosting ensemble can achieve competitive predictive performance (94.83% accuracy on HFGDD) while maintaining transparency, though on the challenging PIDD, its accuracy (76.19%) is below that of some non-interpretable models. Further validation with clinician studies and more diverse base models is needed to fully assess the trade-off.

## Figures and Tables

**Table 1:** Summary of related machine learning research on diabetes prediction and its methodological drawbacks that guide the present study.

Authors [Ref. No.]	Techniques Used	Dataset	% Performance	Major Findings	Limitations
Gupta et al. [29]	Advanced Neural Network (NN)	PIMA (768 cases)	Accuracy: 84.42%	Proposed NN method outperformed conventional ML and ensemble methods with tuned hyperparameters.	Not interpretable; black-box approach, not practical for clinical use.
Bhoi [30]	Logistic Regression (LR), SVM, etc.	PIMA	Accuracy: 76.80% (LR)	LR outperformed other conventional methods.	No explanation capabilities; black box models.
Qin et al. [31]	CATBoost, LR, SVM, XGBoost, RF	PIMA	Accuracy: 82.1% (CATBoost)	The best results were obtained with CATBoost.	Models are black boxes; no explanation for the predictions.
Kalagotla et al. [32]	Stacking Ensemble (MLP, LR, SVM)	PIMA	Accuracy: 78.2%	Heterogeneous stacking ensemble gave better results than the homogeneous AdaBoost.	Stacked architecture compounds interpretability challenges; final prediction aggregates multiple opaque models.
Chang et al. [41]	J48 DT, Naive Bayes (NB), RF	PIMA	Accuracy: 79.13% (RF)	RF was the most accurate among the evaluated algorithms.	No interpretability mechanisms were employed.
Ramesh et al. [42]	SVM with feature processing	PIMA	Accuracy: 83.20%	Feature scaling, augmentation, and selection improved SVM performance.	Black-box approach without explanation of model decisions.
Ahmed et al. [43]	LR, DT, NB, RF	PIMA	Accuracy: 80% (RF)	RF was more accurate, precise, and sensitive than LR, NB, and DT.	No focus on model interpretability or clinical explainability.
Butt et al. [44]	RF, LR, MLP, LSTM	PIMA	Accuracy: 87.26% (LSTM)	LSTM and MLP demonstrated high accuracy, showcasing deep learning potential.	Black-box deep learning models with no inherent interpretability.
Mansouri et al. [45]	Optimized K-Nearest Neighbors (KNN)	PIMA	Accuracy: 76%	Optimized KNN achieved reasonable accuracy with standard preprocessing.	Interpretability is limited to the algorithm’s basic mechanics; no clinical explanations.
Iparraguirre-Villanueva et al. [46]	KNN, LR, DT, NB, SVM	PIMA	Accuracy: 79.6% (KNN)	KNN and NB achieved superior accuracy among classical algorithms.	Prioritized predictive performance while neglecting interpretability.
Islam et al. [58]	Gradient Boosting + SHAP	PIMA	Accuracy: 82.3%	SHAP identified glucose, BMI, and age as dominant risk factors.	Ensemble remains a black box; SHAP applied post hoc, which may not reflect true model reasoning.
Deng et al. [64]	Attention-based Deep Learning	PIMA	Accuracy: 78.5%	Attention weights provided intrinsic feature importance.	Performance is lower than state-of-the-art black-box ensembles.

**Table 2:** Interpretability features of some diabetes prediction studies and positioning of this study.

Study	Interpretability Approach	Intrinsic/Post Hoc	Clinical Validation	Key Limitation (Interpretability)
Gupta et al. [29]	None	–	No	Neural network as black box with no explanation
Kalagotla et al. [32]	None	–	No	Stacking ensemble compounds opacity; no interpretability in fusion
Islam et al. [58]	SHAP (post hoc)	Post hoc	Limited	Explanations may not reflect true model reasoning
Deng et al. [64]	Attention weights	Intrinsic	No	Less accurate than black box ensembles
This Work	Geometric distance + fuzzy membership + SHAP	Intrinsic + post hoc	Clinical discussion	No trade-off between accuracy and interpretability—offers multi-level faithful explanations and delivers competitive accuracy

Note: Performance on PIDD (76.19% accuracy) reflects dataset challenges; HFGDD achieves 94.83%. While MMFDE achieves lower precision (62.04%) on PIDD compared to some base models, its recall (82.72%) is substantially higher—a clinically acceptable trade-off for screening applications where detecting true diabetic cases is paramount.

**Table 3:** Description and statistical range of clinical attributes from the HFGDD and PIDDs.

Attributes	Description	Values
Pregnancies	Pregnancy count	0–17
Glucose	Glucose of plasma, two hours after oral glucose tolerance test (OGTT)	0–199 mg/dL
Blood pressure	Diastolic pressure (mmHg)	0–122 mmHg
Skin thickness	Triceps skinfold thickness (mm)	0–99 mm
Insulin	Serum insulin after 2 h (μU/mL)	0–846 μU/mL
Body mass index (BMI)	Body Mass Index (kg/m^2^)	0–80.6 kg/m^2^
Diabetes inheritance metric (DPF)	Familial history of diabetes	0.078–2.42
Age	Years of age	1–120
Outcome	Diagnosing Diabetes	0: non-diabetic, 1: diabetic

**Table 4:** Hyperparameters of the four base classifiers in the MMFDE framework.

Model	n_Estimators	Learning_Rate	Max_Depth	Regularization
LightGBM	200	0.05	6	L1 = 0.1, L2 = 0.1
XGBoost	200	0.05	6	L1 = 0.1, L2 = 0.1
Gradient Boosting	200	0.05	5	subsample = 0.8
AdaBoost	200	0.05	—	—

**Table 5:** Numerical demonstration of the scores of the base classifier confidence, distance, fuzzy membership, and ultimate prediction with interpretable annotations.

Sample No	1	2	3	4	5
Actual Class	0	0	0	0	1
Base Model Probabilities					
LightGBM	0.0118	0.0931	0.3406	0.1175	0.7920
XGBoost	0.0104	0.0410	0.3200	0.0629	0.6680
GBM	0.0073	0.0747	0.3291	0.5170	0.6115
AdaBoost	0.1277	0.4521	0.0826	0.2896	0.5948
Distances to Ideal Diabetic (D_Pos)	0.5688	0.5672	0.3544	0.4960	0.1264
Distances to Ideal Non-Diabetic (D_Neg)	0.0060	0.0429	0.2333	0.0668	0.4888
Fuzzy Membership Scores					
μ_pos (Diabetic)	0.3383	0.3394	0.5090	0.3887	0.7860
μ_neg (non-diabetic)	0.9887	0.9214	0.6411	0.8805	0.3941
Fuzzy Score	0.3429	0.3585	0.4670	0.3795	0.5967
Confidence Level	Very High	High	Moderate	High	High
Final Prediction	0	0	0	0	1
Correct?	✓	✓	✓	✓	✓

**Table 6:** The confidence classification of the samples in Table 3, according to the thresholds under Section 3.6.3.

Sample	μ_max	μ_max Value	Threshold Range	Confidence Level
1	μ_neg	0.9887	>0.9	Very High
2	μ_neg	0.9214	>0.9	Very High
3	μ_neg	0.6411	≤0.7	Moderate
4	μ_neg	0.8805	0.7 < μ ≤ 0.9	High
5	μ_pos	0.7860	0.7 < μ ≤ 0.9	High

**Table 7:** Results of proposed multi-metric fuzzy distance-based ensemble and baseline models using two diabetes datasets.

Dataset	Model	Accuracy	Precision	Recall	F1-Score	AUC
HFGDD	LightGBM	92.33	87.68	90.24	88.94	96.28
	GBM	94.67	91.79	92.68	92.23	96.71
	XGBoost	93.67	89.95	91.71	90.82	96.71
	AdaBoost	75.83	68.75	53.66	60.27	85.30
	MMFDE (Proposed)	94.83	89.19	96.59	92.74	97.66
PIDD	LightGBM	74.89	67.16	55.56	60.81	82.38
	GBM	75.76	69.84	54.32	61.11	83.21
	XGBoost	73.16	82.76	29.63	43.64	81.08
	AdaBoost	74.89	70.18	49.38	57.97	83.56
	MMFDE (Proposed)	76.19	62.04	82.72	70.90	84.12

**Table 8:** Statistical significance results (*p*-values) comparing MMFDE with base models.

Comparison	HFGDD Accuracy	HFGDD AUC	PIDD Accuracy	PIDD AUC
MMFDE vs. LightGBM	0.012	0.008	0.023	0.015
MMFDE vs. GBM	0.041	0.032	0.038	0.027
MMFDE vs. XGBoost	0.018	0.021	0.009	0.011
MMFDE vs. AdaBoost	<0.001	<0.001	<0.001	<0.001

**Table 9:** Confidence level distribution across predictions.

Confidence Level	Threshold	HFGDD (% of Predictions)	PIDD (% of Predictions)
Very High	μ_max > 0.9	78.3%	52.1%
High	0.7 < μ_max ≤ 0.9	16.5%	31.4%
Moderate	μ_max ≤ 0.7	5.2%	16.5%

**Table 10:** Quantitative concordance between geometric distances and SHAP attributions on test sets.

Dataset	Concordance (%)	Discordance (%)	% of Discordant Cases with μ_max ≤ 0.7	Misclassification Rate in Concordant Cases (%)	Misclassification Rate in Discordant Cases (%)
**HFGDD**	92.7	7.3	68%	3.1	18.4
**PIDD**	84.5	15.5	72%	9.2	29.5

**Table 11:** Ablation study results on HFGDD.

Configuration	Accuracy (%)	AUC (%)	Δ Accuracy
Full MMFDE (all 4 metrics)	94.83	97.66	-
Without Euclidean	93.67	96.52	−1.16
Without Manhattan	93.92	96.71	−0.91
Without Cosine	94.25	97.11	−0.58
Without Chebyshev	94.33	97.18	−0.50

**Table 12:** Comparison with state-of-the-art methods for predicting diabetes.

Study	Method	Dataset	Preprocessing/PIDD Version	Accuracy (%)	AUC (%)	Interpretability Provided
Gupta et al. [29]	Deep NN	PIDD	Zero imputation	84.42	–	None
Kalagotla et al. [32]	Stacking Ensemble	PIDD	Zero imputation	78.20	–	None
Butt et al. [36]	LSTM	PIDD	Zero imputation	87.26	–	None
Ahmed et al. [35]	RF	PIDD	Zero imputation	80.00	–	Feature importance only
Islam et al. [58]	Gradient Boosting + SHAP	PIDD	Mean imputation	82.30	–	Post hoc SHAP
Deng et al. [48]	Attention-based DL	PIDD	Zero imputation	78.50	–	Attention weights
This Work	MMFDE + SHAP	PIDD	Zero imputation (with physiological constraints)	76.19	84.12	Geometric + Fuzzy + SHAP (Intrinsic + post hoc)
This Work	MMFDE + SHAP	HFGDD	No missing values	94.83	97.66	Geometric + Fuzzy + SHAP (Intrinsic + post hoc)

Footnote: Direct accuracy comparisons across studies should consider differences in preprocessing (e.g., handling of zero values in PIDD) and train/test splits. Our PIDD results use zero imputation but with physiological constraints (e.g., zero glucose treated as missing and imputed). HFGDD had no missing values.

## Data Availability

The original data presented in the study are openly available in [32,33].

## Supplementary Materials

The following supporting information can be downloaded at: https://www.mdpi.com/article/10.3390/diagnostics16091254/s1.

## References

[1] Boadu A.A., Yeboah-Manu M., Osei-Wusu S., Yeboah-Manu D. (2024). Tuberculosis and diabetes mellitus: The complexity of the comorbid interactions. Int. J. Infect. Dis..

[2] Kiran M., Xie Y., Anjum N., Ball G., Pierscionek B., Russell D. (2025). Machine learning and artificial intelligence in type 2 diabetes prediction: A comprehensive 33-year bibliometric and literature analysis. Front. Digit. Health.

[3] Metwally A.A., Perelman D., Park H., Wu Y., Jha A., Sharp S., Celli A., Ayhan E., Abbasi F., Gloyn A.L. (2025). Prediction of metabolic subphenotypes of type 2 diabetes via continuous glucose monitoring and machine learning. Nat. Biomed. Eng..

[4] Nandiraju S.K.K., Chundru S.K., Vangala S.R., Polam R.M., Kamarthapu B., Kakani A.B. (2025). Towards Early Forecast of Diabetes Mellitus via Machine Learning Systems in Healthcare. Eur. J. Technol..

[5] Carmichael J., Fadavi H., Ishibashi F., Shore A.C., Tavakoli M. (2021). Advances in screening, early diagnosis and accurate staging of diabetic neuropathy. Front. Endocrinol..

[6] Tiwari D., Aw T.C. (2024). The 2024 American Diabetes Association guidelines on standards of medical care in diabetes: Key takeaways for laboratory. Explor. Endocr. Metab. Dis..

[7] Davidson K.W., Barry M.J., Mangione C.M., Cabana M., Caughey A.B., Davis E.M., Donahue K.E., Doubeni C.A., Krist A.H., Kubik M. (2021). Screening for prediabetes and type 2 diabetes: US Preventive Services Task Force recommendation statement. JAMA.

[8] Zhang J., Zhang Z., Zhang K., Ge X., Sun R., Zhai X. (2023). Early detection of type 2 diabetes risk: Limitations of current diagnostic criteria. Front. Endocrinol..

[9] Afsaneh E., Sharifdini A., Ghazzaghi H., Ghobadi M.Z. (2022). Recent applications of machine learning and deep learning models in the prediction, diagnosis, and management of diabetes: A comprehensive review. Diabetol. Metab. Syndr..

[10] Almutairi E., Abbod M., Hunaiti Z. (2025). Prediction of Diabetes Using Statistical and Machine Learning Modelling Techniques. Algorithms.

[11] Templer S., Abdo S., Wong T. (2024). Preventing diabetes complications. Intern. Med. J..

[12] Shapiro M.R., Tallon E.M., Brown M.E., Posgai A.L., Clements M.A., Brusko T.M. (2025). Leveraging artificial intelligence and machine learning to accelerate discovery of disease-modifying therapies in type 1 diabetes. Diabetologia.

[13] Jiang H., Wang H., Pan T., Liu Y., Jing P., Liu Y. (2024). Mobile application and machine learning-driven scheme for intelligent diabetes progression analysis and management using multiple risk factors. Bioengineering.

[14] Khalifa M., Albadawy M. (2024). Artificial intelligence for diabetes: Enhancing prevention, diagnosis, and effective management. Comput. Methods Programs Biomed. Update.

[15] Guan Z., Li H., Liu R., Cai C., Liu Y., Li J., Wang X., Huang S., Wu L., Liu D. (2023). Artificial intelligence in diabetes management: Advancements, opportunities, and challenges. Cell Rep. Med..

[16] Hu P., Li X., Lu N., Dong K., Bai X., Liang T., Li J. (2023). Prediction of new-onset diabetes after pancreatectomy with subspace clustering based multi-view feature selection. IEEE J. Biomed. Health Inform..

[17] Yenurkar G.K., Mal S., Nyangaresi V.O., Hedau A., Hatwar P., Rajurkar S., Khobragade J. (2023). Multifactor data analysis to forecast an individual’s severity over novel COVID-19 pandemic using extreme gradient boosting and random forest classifier algorithms. Eng. Rep..

[18] Yenurkar G.K., Mal S. (2022). Effective detection of COVID-19 outbreak in chest X-Rays using fusionnet model. Imaging Sci. J..

[19] Wajgi R., Yenurkar G., Nyangaresi V.O., Wanjari B., Verma S., Deshmukh A., Mallewar S. (2024). Optimized tuberculosis classification system for chest X-ray images: Fusing hyperparameter tuning with transfer learning approaches. Eng. Rep..

[20] Yenurkar G.K., Mal S., Thakur N., Dhomne S., Dhurve M., Patel M., Kulmeti K., Dhurve H. (2025). DeepLeuk: A convolutional neural network pre-trained model for microscopic cell images-Based leukemia cancer analysis. Multimed. Tools Appl..

[21] Yenurkar G.K., Mal S., Wakulkar A., Umbarkar K., Bhat A., Bhasharkar A., Pathade A. (2024). Future prediction for precautionary measures associated with heart-related issues based on IoT prototype. Multimed. Tools Appl..

[22] Kale Y., Rathkanthiwar S., Yenurkar G., Mal S., Nyangaresi V.O., Kamble S., Damahe L., Bankar N. (2024). Revolutionizing chronic heart disease management: The role of IoT-based ambulatory blood pressure monitoring system. Diagnostics.

[23] Khekare G., Yenurkar G., Turukmane A.V., Ameta G.K., Sharma P., Phulre A.K. (2024). Artificial intelligence algorithms for better decision-making. Multi-Criteria Decision-Making and Optimum Design with Machine Learning.

[24] Almulihi A., Saleh H., Hussien A.M., Mostafa S., El-Sappagh S., Alnowaiser K., Ali A.A., Refaat Hassan M. (2022). Ensemble learning based on hybrid deep learning model for heart disease early prediction. Diagnostics.

[25] Mahajan P., Uddin S., Hajati F., Moni M.A. (2023). Ensemble Learning for Disease Prediction: A Review. Healthcare.

[26] Livieris I.E., Kanavos A., Tampakas V., Pintelas P.E. (2019). A Weighted Voting Ensemble Self-Labeled Algorithm for the Detection of Lung Abnormalities from X-Rays. Algorithms.

[27] Livieris I.E., Kanavos A., Tampakas V., Pintelas P.E. (2018). An Ensemble SSL Algorithm for Efficient Chest X-Ray Image Classification. J. Imaging.

[28] Majumder A.B., Gupta S., Singh D., Acharya B., Gerogiannis V.C., Kanavos A., Pintelas P.E. (2023). Heart Disease Prediction Using Concatenated Hybrid Ensemble Classifiers. Algorithms.

[29] Gupta N., Kaushik B., Imam Rahmani M.K., Lashari S.A. (2023). Performance Evaluation of Deep Dense Layer Neural Network for Diabetes Prediction. Comput. Mater. Contin..

[30] Bhoi S.K., Panda S.K., Jena K.K., Abhisekh P.A., Sahoo K.S., Sama N.U., Pradhan S.S., Sahoo R.R. (2021). Prediction of diabetes in females of pima Indian heritage: A complete supervised learning approach. Turk. J. Comput. Math. Educ..

[31] Qin Y., Wu J., Xiao W., Wang K., Huang A., Liu B., Yu J., Li C., Yu F., Ren Z. (2022). Machine learning models for data-driven prediction of diabetes by lifestyle type. Int. J. Environ. Res. Public Health.

[32] Kalagotla S.K., Gangashetty S.V., Giridhar K. (2021). A novel stacking technique for prediction of diabetes. Comput. Biol. Med..

[33] Lundberg S.M., Lee S.-I. (2017). A Unified Approach to Interpreting Model Predictions. Adv. Neural Inf. Process. Syst..

[34] Ribeiro M.T., Singh S., Guestrin C. (2016). “Why Should I Trust You?”: Explaining the Predictions of Any Classifier. Proceedings of the 22nd ACM SIGKDD International Conference on Knowledge Discovery and Data Mining (KDD ‘16).

[35] Rudin C. (2019). Stop explaining black box machine learning models for high stakes decisions and use interpretable models instead. Nat. Mach. Intell..

[36] Lakkaraju H., Arsov N., Bastani O. (2020). Robust and Stable Black Box Explanations. Proc. Mach. Learn. Res..

[37] Doshi-Velez F., Kim B. (2017). Towards a rigorous science of interpretable machine learning. arXiv.

[38] Molnar C. (2022). Interpretable Machine Learning: A Guide for Making Black Box Models Explainable. https://christophm.github.io/interpretable-ml-book/.

[39] Ahmad M.A., Eckert C., Teredesai A. (2018). Interpretable Machine Learning in Healthcare. Proceedings of the 2018 ACM International Conference on Bioinformatics, Computational Biology, and Health Informatics (BCB ‘18).

[40] Stiglic G., Kocbek P., Fijacko N., Zitnik M., Verbert K., Cilar L. (2020). Interpretability of machine learning-based prediction models in healthcare. WIREs Data Min. Knowl. Discov..

[41] Chang V., Bailey J., Xu Q.A., Sun Z. (2023). Pima indians diabetes mellitus classification based on machine learning (ML) algorithms. Neural Comput. Appl..

[42] Ramesh J., Aburukba R., Sagahyroon A. (2021). A remote healthcare monitoring framework for diabetes prediction using machine learning. Healthc. Technol. Lett..

[43] Ahmed A., Khan J., Arsalan M., Ahmed K., Shahat A.A., Alhalmi A., Naaz S. (2025). Machine Learning Algorithm-Based Prediction of Diabetes Among Female Population Using PIMA Dataset. Healthcare.

[44] Butt U.M., Letchmunan S., Ali M., Hassan F.H., Baqir A., Sherazi H.H.R. (2021). Machine learning based diabetes classification and prediction for healthcare applications. J. Healthc. Eng..

[45] Mansouri S., Boulares S., Chabchoub S. (2024). Machine Learning for Early Diabetes Detection and Diagnosis. J. Wirel. Mob. Netw. Ubiquitous Comput. Dependable Appl..

[46] Iparraguirre-Villanueva O., Espinola-Linares K., Castañeda R.O.F., Cabanillas-Carbonell M. (2023). Application of Machine Learning Models for Early Detection and Accurate Classification of Type 2 Diabetes. Diagnostics.

[47] Breiman L. (2001). Random forests. Mach. Learn..

[48] Friedman J.H. (2001). Greedy function approximation: A gradient boosting machine. Ann. Stat..

[49] Wolpert D.H. (1992). Stacked generalization. Neural Netw..

[50] Guidotti R., Monreale A., Ruggieri S., Turini F., Giannotti F., Pedreschi D. (2018). A survey of methods for explaining black box models. ACM Comput. Surv..

[51] Lakkaraju H., Bastani O. (2020). “How do I fool you?” Manipulating user trust via misleading black box explanations. Proceedings of the AAAI/ACM Conference on AI, Ethics, and Society.

[52] Letham B., Rudin C., McCormick T.H., Madigan D. (2015). Interpretable classifiers using rules and Bayesian analysis: Building a better stroke prediction model. Ann. Appl. Stat..

[53] Bahdanau D., Cho K., Bengio Y. (2014). Neural machine translation by jointly learning to align and translate. arXiv.

[54] Lou Y., Caruana R., Gehrke J. (2012). Intelligible models for classification and regression. Proceedings of the 18th ACM SIGKDD International Conference on Knowledge Discovery and Data Mining.

[55] Freitas A.A. (2014). Comprehensible classification models: A position paper. ACM SIGKDD Explor. Newsl..

[56] Holzinger A., Langs G., Denk H., Zatloukal K., Müller H. (2019). Causability and explainability of artificial intelligence in medicine. WIREs Data Min. Knowl. Discov..

[57] Adadi A., Berrada M. (2018). Peeking inside the black-box: A survey on explainable artificial intelligence (XAI). IEEE Access.

[58] Islam M.M., Rifat H.R., Shahid M.S.B., Akhter A., Uddin M.A., Uddin K.M.M. (2024). Explainable machine learning for efficient diabetes prediction using hyperparameter tuning, SHAP analysis, partial dependency, and LIME. Eng. Rep..

[59] Wachter S., Mittelstadt B., Russell C. (2017). Counterfactual explanations without opening the black box: Automated decisions and the GDPR. Harv. J. Law Technol..

[60] Tibshirani R. (1996). Regression shrinkage and selection via the lasso. J. R. Stat. Soc. Ser. B.

[61] Caruana R., Lou Y., Gehrke J., Koch P., Sturm M., Elhadad N. (2015). Intelligible models for healthcare: Predicting pneumonia risk and hospital 30-day readmission. Proceedings of the 21th ACM SIGKDD International Conference on Knowledge Discovery and Data Mining.

[62] Kopitar L., Kocbek P., Cilar L., Sheikh A., Stiglic G. (2020). Early detection of type 2 diabetes mellitus using machine learning-based prediction models. Sci. Rep..

[63] Ellahham S. (2020). Artificial intelligence: The future for diabetes care. Am. J. Med..

[64] Deng X., Huang D., Jia L., Zhu Y., Wang L. An interpretable deep learning model for diabetes prediction. Proceedings of the 2020 IEEE International Conference on Bioinformatics and Biomedicine (BIBM).

[65] Tjoa E., Guan C. (2020). A survey on explainable artificial intelligence (XAI): Toward medical XAI. IEEE Trans. Neural Netw. Learn. Syst..

[66] Jiang X., Osl M., Kim J., Ohno-Machado L. (2012). Calibrating predictive model estimates to support personalized medicine. J. Am. Med. Inform. Assoc..

[67] Tonekaboni S., Joshi S., McCradden M.D., Goldenberg A. (2019). What clinicians want: Contextualizing explainable machine learning for clinical end use. Proc. Mach. Learn. Res..

[68] Vellido A. (2020). The importance of interpretability and visualization in machine learning for applications in medicine and health. Neural Comput. Appl..

[69] Hospital Frankfurt Germany Diabetes Dataset. https://www.kaggle.com/johndasilva/diabetes.

[70] Pima Indians Diabetes Database. https://www.kaggle.com/datasets/uciml/pima-indians-diabetes-database.

[71] Nti I.K., Nyarko-Boateng O., Aning J. (2021). Performance of Machine Learning Algorithms with Different K Values in K-fold CrossValidation. Int. J. Inf. Technol. Comput. Sci..

[72] Ke G., Meng Q., Finley T., Wang T., Chen W., Ma W., Ye Q., Liu T.Y. (2017). LightGBM: A highly efficient gradient boosting decision tree. Adv. Neural Inf. Process. Syst..

[73] Ju Y., Sun G., Chen Q., Zhang M., Zhu H., Rehman M.U. (2019). A model combining convolutional neural network and LightGBM algorithm for ultra-short-term wind power forecasting. IEEE Access.

[74] Wang Y., Wang T. (2020). Application of improved LightGBM model in blood glucose prediction. Appl. Sci..

[75] Chen T., Guestrin C. (2016). XGBoost: A scalable tree boosting system. Proceedings of the 22nd ACM SIGKDD International Conference on Knowledge Discovery and Data Mining.

[76] Chen T., He T., Benesty M., Khotilovich V., Tang Y., Cho H., Chen K., Mitchell R., Cano I., Zhou T. (2015). Xgboost: Extreme Gradient Boosting.

[77] Freund Y., Schapire R.E. (1997). A decision-theoretic generalization of on-line learning and an application to boosting. J. Comput. Syst. Sci..

[78] Liberti L., Lavor C., Maculan N., Mucherino A. (2014). Euclidean distance geometry and applications. SIAM Rev..

[79] Craw S., Sammut C., Webb G.I. (2017). Manhattan Distance. Encyclopedia of Machine Learning and Data Mining.

[80] Cantrell C.D. (2000). Modern Mathematical Methods for Physicists and Engineers.

[81] Nayak S., Bhat M., Subba Reddy N.V., Ashwath Rao B. (2022). Study of distance metrics on k-nearest neighbor algorithm for star categorization. J. Phys. Conf. Ser..

[82] Fawcett T. (2006). An introduction to ROC analysis. Pattern Recognit. Lett..

[83] Saito T., Rehmsmeier M. (2015). The precision-recall plot is more informative than the ROC plot when evaluating binary classifiers on imbalanced datasets. PLoS ONE.

[84] Dietterich T.G. (1998). Approximate statistical tests for comparing supervised classification learning algorithms. Neural Comput..

