# Supplementary material for: An Interpretable Fuzzy Distance-Based Ensemble Framework with SHAP Analysis for Clinically Transparent Prediction of Diabetes
Source: Diagnostics (Basel). 2026 Apr 22;16(9):1254. doi: 10.3390/diagnostics16091254 (PMC13162795; doi:10.3390/diagnostics16091254)
Supplement: Supplementary file 1 [file diagnostics-16-01254-s001.zip › diagnostics-4204362-supplementary.pdf]

Supplementary File S1

An Interpretable Fuzzy Distance-Based Ensemble Framework with SHAP Analysis for Clinically Transparent Prediction of diabetes.

Asif Hassan Syed[3], Altyeb Altaher Taha[1,*], Ahmed Hamza Osman[2], Yakubu Suleiman Baguda[2], Hani Moaiteq Aljahdali[2], and Arda Yunianta [2],

Section S1 (Supplementary Materials): Detailed Decision Walkthrough for a Representative Case

To illustrate how the proposed MMFDE framework arrives at a prediction and provides multi-level interpretability, we present a step-by-step walkthrough for Sample 5 from Table 3. This case was correctly classified as diabetic with high confidence ($\mu\_max = \mu\_pos = 0.7860$). The walkthrough follows the sequential flow described in Sections 3.6 and 3.7 and shows how each technical component translates into clinically meaningful interpretation.

S1.1. Input Features and Base Classifier Outputs

The patient's clinical measurements (HFGDD test set) are:

Feature	Value
Glucose	148 mg/dL
BMI	33.2 kg/m^2
Age	58 years
DPF	0.98
Blood pressure	78 mmHg
Skin thickness	35 mm
Insulin	0 µU/mL
Pregnancies	2

These values are preprocessed (min-max scaled to [0,1]) and fed into the four base classifiers (LightGBM, XGBoost, GBM, AdaBoost). Their predicted probabilities for the diabetic class are:

Classifier	Probability (diabetic)
LightGBM	0.7920
XGBoost	0.6680
GBM	0.6115
AdaBoost	0.5948

All four classifiers agree on a diabetic prediction, though with varying confidence.

S1.2. Distance to Ideal Reference Vectors

For class *diabetic*, the ideal confidence vector is $[1, 1, 1, 1]$ (all classifiers 100% certain). For class *non-diabetic*, the ideal vector is $[0, 0, 0, 0]$. The prediction vector for Sample 5 is $p = [0.7920, 0.6680, 0.6115, 0.5948]$.

Using the weighted hybrid distance (weights: Euclidean 30%, Manhattan 30%, Cosine 20%, Chebyshev 20%), the distances to the ideal vectors are computed (see Table 3):

- **Distance to ideal diabetic (D_{Pos})** = 0.1264
- **Distance to ideal non-diabetic (D_{Neg})** = 0.4888

***Interpretation*:** The prediction vector is much closer to the diabetic ideal (0.1264) than to the non-diabetic ideal (0.4888), indicating strong alignment with the diabetic archetype.

S1.3. Fuzzy Membership and Confidence Calibration

Distances are converted to fuzzy membership values using an exponential decay function with optimized β:

- $\boldsymbol{\mu_{pos} = e^{-\beta D_{Pos}}} = 0.7860$
- $\boldsymbol{\mu_{neg} = e^{-\beta D_{Neg}}} = 0.3941$

The fuzzy membership scores represent calibrated confidence: $\mu_{pos} = 0.7860$ indicates high confidence in the diabetic prediction. According to the thresholds defined in Section 3.6.3, this falls into the High Confidence category ($0.7 < \mu \leq 0.9$), appropriate for clinical decision support with basic verification.

S1.4. Final Prediction

The normalized fuzzy score is:

$$\textbf{Fuzzy score} = \frac{e^{\mu_{pos}}}{e^{\mu_{pos}} + e^{\mu_{neg}}} = 0.5967$$

Since this exceeds 0.5, the final prediction is diabetic (class 1), matching the ground truth.

S1.5. Feature-Level Explanation (SHAP Force Plot)

The SHAP force plot for Sample 5 (Figure 11 in the main text) shows the contribution of each clinical feature to the final prediction. Positive contributions (red) push toward a diabetic prediction; negative contributions (blue) push toward non-diabetic.

- **Glucose (+0.48):** Strongest positive contribution, consistent with elevated plasma glucose.
- **BMI (+0.31):** Second strongest positive contribution, reflecting obesity.
- **Age (+0.08):** Mild positive contribution.
- **DPF (+0.05):** Small positive contribution.
- **Blood pressure (–0.02):** Slightly negative (protective) contribution.

The force plot explains that the patient’s high glucose and BMI are the primary drivers of the diabetic prediction, while other features have marginal influence.

S1.6. Integrated Interpretability Summary

For Sample 5, the framework provides:

- **Geometric interpretability:** The prediction vector is 0.1264 units from the diabetic ideal and 0.4888 from the non-diabetic ideal – a simple, intuitive measure of how “typical” the case is.
- **Confidence-calibrated interpretability:** A fuzzy membership of 0.7860 (High Confidence) tells the clinician that the model is reasonably certain, but verification may be prudent.
- **Feature-level interpretability:** The SHAP force plot shows that glucose and BMI are the main drivers, aligning with established diabetes risk factors and enabling patient-specific discussion.

This walkthrough demonstrates that MMFDE's decisions are not only accurate but also transparent at multiple levels, supporting clinical trust and informed decision-making.
